# Increasing returns to scale: The solution to the second-order social dilemma

**DOI:** 10.1038/srep31927

**Published:** 2016-08-18

**Authors:** Hang Ye, Shu Chen, Jun Luo, Fei Tan, Yongmin Jia, Yefeng Chen

**Affiliations:** 1 College of Economics, Interdisciplinary Center for Social Sciences (ICSS), Zhejiang University, Hangzhou, China; 2 School of Economics, Center for Economic Behavior and Decision-making (CEBD), Zhejiang University of Finance and Economics, Hangzhou, China; 3 Department of Economics, Saint Louis University, St.Louis, USA

## Abstract

Humans benefit from extensive cooperation; however, the existence of free-riders may cause cooperation to collapse. This is called the social dilemma. It has been shown that punishing free-riders is an effective way of resolving this problem. Because punishment is costly, this gives rise to the second-order social dilemma. Without exception, existing solutions rely on some stringent assumptions. This paper proposes, under very mild conditions, a simple model of a public goods game featuring increasing returns to scale. We find that punishers stand out and even dominate the population provided that the degree of increasing returns to scale is large enough; consequently, the second-order social dilemma dissipates. Historical evidence shows that people are more willing to cooperate with others and punish defectors when they suffer from either internal or external menaces. During the prehistoric age, the abundance of contributors was decisive in joint endeavours such as fighting floods, defending territory, and hunting. These situations serve as favourable examples of public goods games in which the degrees of increasing returns to scale are undoubtedly very large. Our findings show that natural selection has endowed human kind with a tendency to pursue justice and punish defection that deviates from social norms.

It was only approximately 12,000 years ago, with the emergence of agricultural civilization in Mesopotamia, that individuals and families alike were able to play primary roles in economic activities^1^. In other words, in more than 99% of the 7 million years of human evolutionary history^2^, our ancestors had to rely on joint endeavours rather than individuals or families to maintain species survival. This life pattern includes joint projects such as hunting large game, fighting natural disasters, defending territory against invaders, and, most importantly, sharing the fruits of these cooperative activities^3^,^4^,^5^. This life pattern can be viewed as a particular mechanism for producing and distributing public goods.

Public goods games (PGGs) have been widely employed to investigate the evolution of human cooperation within the framework of evolutionary game theory. In a standard PGG, the total return of a joint project is divided equally among all participants, which means that a defector can free-ride other participants and hence can fare better than others. This situation raises an evolutionary puzzle: how do cooperators rise and evolve? Because the payoff of a cooperator is strictly lower than that of a defector, each participant, in light of the hypothesis of self-interest, chooses to contribute nothing to the PGG in Nash equilibrium. As a result, any individual’s payoff will be zero. This is called the social dilemma^6^,^7^.

It is widely believed that punishing defection serves as an effective way to resolve this problem. Assume that some players, called punishers or altruistic punishers, have a strong sense of justice and not only contribute but also punish those who deviate from social norms. If the strength of punishment exceeds the contribution cost, free-riding becomes unprofitable, and the PGG is immune from the social dilemma^8^,^9^,^10^,^11^,^12^,^13^. Related anthropological and zoological studies show that punishing defection was not only an important way for human beings to maintain cooperation in early societies^14^,^15^ but is also a vital means for many types of animals to maintain species survival^16^,^17^.

However, punishment is costly because it takes time and energy and invites retaliation. As a result, the payoff of a punisher is lower than that of a cooperator. In light of the hypothesis of self-interest, each participant will choose to co-operate but not punish in Nash equilibrium. These cooperators are called second-order free-riders to distinguish them from defectors. This situation presents a second evolutionary puzzle for humankind: how do punishers rise and evolve? Once punishers are invaded by cooperators, defectors can easily invade and dominate the population; thus, defection eventually prevails in the evolutionary equilibrium. This is called the second-order social dilemma^8^,^18^,^19^,^20^.

From the perspective of the evolution of simulation, there are two main types of relevant studies to solve the problem of the second-order social dilemma. One type is based on the viewpoint of the social network^21^,^22^,^23^,^24^, including different mechanisms of punishment, such as adaptive punishment^25^, probabilistic punishment^26^,^27^, and conditional punishment^28^. Reward is another solution to the second-order social dilemma^29^. The use of a combination of rewards and punishment is also an important method^30^,^31^,^32^,^33^. The evolutionary games of spatial networks not only provide an important perspective for solving the problem of the first-order social dilemma^34^,^35^ but also shed light on the solution for the second-order social dilemma.

The second categorization relates to the agent-based model, but none of these models is immune from some strong conditions: (i) external conditions, including the group selection effect resulting from immigration^36^,^37^, indirect reciprocity that is dependent on the reputation mechanism^38^,^39^, and the effect of cultural selection^40^,^41^ or religious indoctrination^42^,^43^; and (ii) internal conditions, including the addition of new strategies or new types of behaviour to alter the payoff matrix of the game, such as voluntary participation^44^,^45^, rewards^46^, sympathy^47^, and pool punishment^48^ or the modification of game rules that may change the nature of the game, such as communication^45^, coordination^49^, and cooperation^50^ among punishers. These strong conditions inevitably narrow our scope of interpretation^51^.

In accordance with the intrinsic properties of human co-operation, we propose a model of PGG without imposing any further assumptions (including new strategies, behaviour types, or game rules) to resolve the second-order social dilemma. It is well known that one of the most important properties of human cooperation is economies of scale^51^,^52^.

As an example, Fig. 1 shows collective hunting in a primitive society. Assume that both the number of the prey per unit area and the length a hunter is able to siege are given. In this case, the hunting returns will depend on the area controlled by the hunters and, ultimately, on the number of hunters. Let *l* denote the length a hunter is able to siege. Then, *n* hunters can siege an area with a circumference of *nl*, and they can control at best an area of (*l*^2^/4*π*)*n*^2^. Therefore, the return of adding one more hunter to this hunting activity grows exponentially. Such a scenario is termed “economies of scale” or “increasing returns to scale”.

According to the aforementioned discussion, we construct a general model of PGG featuring increasing returns to scale. Our analysis of the stochastic evolutionary dynamics and the corresponding computer simulations show that punishers will achieve dominating evolutionary advantages and are able to resist any invasion of second-order free-riders provided that the degree of increasing returns to scale is sufficiently large. Thus, the second-order social dilemma can be effectively resolved.

## Results

### Analytical results

**Figure 2:**
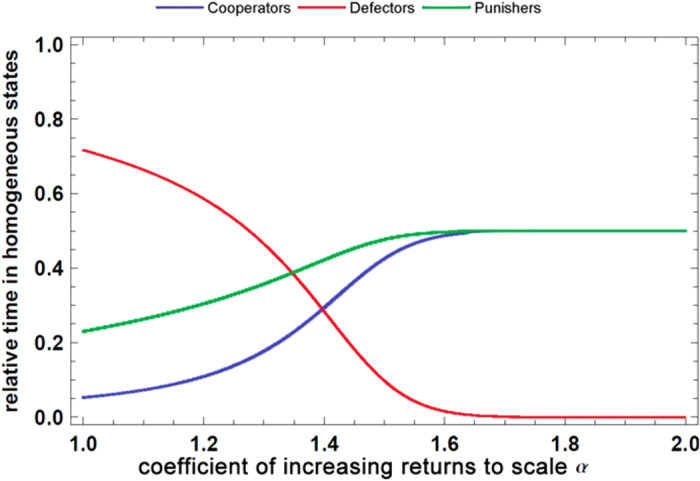
The relative time of strategies in homogenous states. In the calculations, the parameter values are *M* = 100, *N* = 5, *c* = 1, *r* = 3, *δ* = 1, *γ* = 0.3, *ω* = 0.1, *μ* → 0.

We establish a stochastic evolutionary model of PGG in a finite population. To see the effect of an economy of scale, we calculate the relative time of cooperators (*X*), defectors (*Y*) and punishers (*Z*) in homogeneous states as a function of the coefficient of increasing returns to scale (denoted as *α*). The relative time in homogeneous states means the probability of the population being occupied entirely by one of the three strategies. The parameters in the model are population size (*M*), sample size (*N*), contribution cost(*c*), multiplier of return (*r*), strength of punishment (*δ*), cost of punishment (*γ*), selection strength (*ω*) and mutation rate (*μ*). The results are plotted in Fig. 2 below.

As seen in the figure above, in the stochastic evolutionary model, both punishers and cooperators become increasingly dominant relative to defectors if the coefficient of increasing returns to scale *α* ≥ 1.3. Punishers and cooperators even jointly dominate the population and are unlikely to be invaded by defectors when *α* ≥ 1.6. In this case, defectors almost certainly become extinct. Because punishment is no longer necessary, cooperators and punishers fare the same. Consequently, the second-order social dilemma dissipates.

### Simulation results

**Figure 3:**
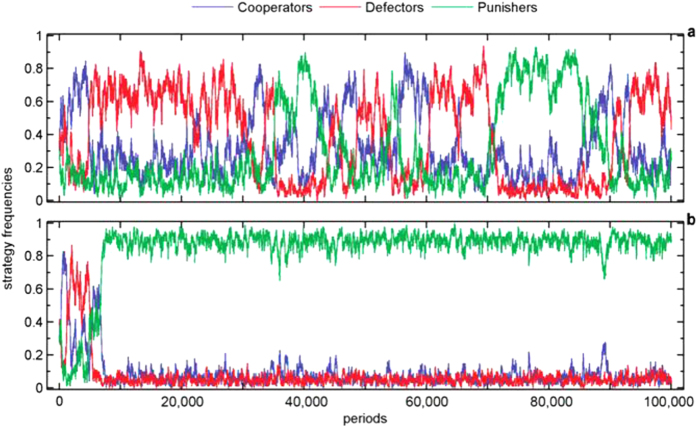
Evolution of cooperation in PGG. In the simulations, the parameter values are *M* = 100, *N* = 5*, X* = 30, *Y* = 40, *Z* = 30, *c* = 1, *r* = 3, *α* = 1.0 or *α* = 1.8, *δ* = 1, *γ* = 0.3, *ω* = 0.5, *μ* = 0.001. (**a**)With constant returns to scale (*α* = 1). (**b**) With increasing returns to scale (*α* = 1.8).

We also adopt a frequency-dependent Moran process to specify the stochastic dynamics of a PGG in a finite population and run a series of multi-agent computer simulations. Our computer simulations show that the punishing strategy cannot gain a foothold in the population if the PGG is of constant returns to scale (*α* = 1). Typically, a rock-paper-scissors-like evolution path governed by the alternating temporary domination of each type will emerge, so the system is unable to form stable cooperation (Fig. 3a). However, punishment becomes the only evolutionarily dominant strategy of the three types in the population provided that the degree of increasing returns to scale of the PGG is sufficiently large (for example, *α* = 1.8). The resulting evolution path shows that after a number of transient oscillations, punishers immediately come to dominate the population and can resist the invasion of any other strategy (Fig. 3b). This implies that the second-order social dilemma dissipates in the PGG featuring increasing returns to scale.

### Robust tests

The simulation results in Fig. 3 are robust when we extend the periods to more than 1 million or change the initial composition of the population. The simulation was repeated 20 times, and all displayed a similar montage. As a result, we randomly chose one as the representative montage of the simulation result.

**Figure 4:**
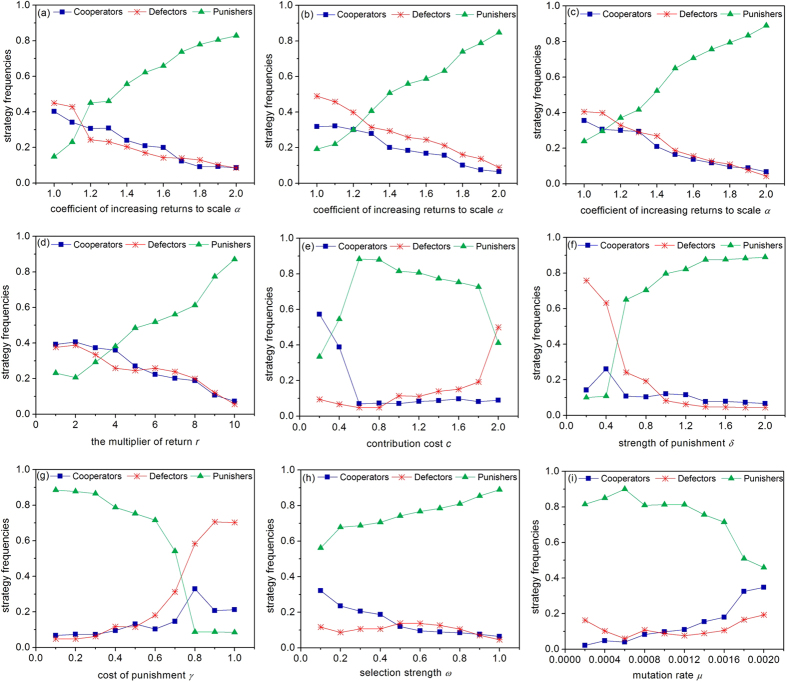
The effect of different parameter values on the evolution of cooperation in a PGG. (**a** ~ **c**) coefficient of increasing returns to scale *α* in different initial composition: (**a**) 100% cooperators, (**b**) 100% defectors, (**c**) 100% punishers; (**d**) multiplier of return *r*; (**e**) contribution cost *c*; (**f**) strength of punishment *δ*; (**g**) cost of punishment *γ*; (**h**) selection strength *ω*; (**i**) mutation rate *μ*. The parameter values are *X* = 30, *Y* = 40, *Z* = 30 (except for figure **a** ~ **c**), *r* = 3 (except for figure **d**), *c* = 1 (except for figure **e**), *α* = 1.0 (except for figure d), *α* = 1.8 (except for figure **a** ~ **c**), *δ* = 1 (except for figure **f**), *γ* = 0.3 (except for figure **g**), *ω* = 0.5 (except for figure **h**), *μ* = 0.001 (except for figure **i**).

To further test the robustness of the results obtained, we studied how different parameter values can affect the evolution of cooperation in a PGG (Fig. 4). The strategy frequency here is the averaged proportion of different strategies in the population in 100,000 periods. All of the results are averaged over 20 times, and they are not affected by the initial composition of the population.

We can see that there is a threshold value of *α* of approximately 1.2 in which the punishers and the defectors are very similar. When *α* is larger than 1.2, the punishers can gradually gain an advantage against both the defectors and the cooperators. The multiplier of return *r* has a similar effect as *α*. Moreover, increasing the cost of contribution and punishment has a negative effect on the maintenance of cooperation, whereas increasing the strength of punishment, the selection strength and the mutation rate helps the punishers in defeating the defectors, which in turn has corresponding effects on the threshold value of *α*. Due to space limitations, other robust tests on various parameters can be found in the Supplementary Materials (Fig. S2–S12).

## Discussion

### The underlying mechanism of increasing returns to scale to solve the second-order social dilemma

Why do punishers fare best in the PGG with increasing returns to scale, hence resolving the second-order social dilemma? It is well known that punishers are primarily threatened by cooperators’ second-order free-riding. However, by analysing the simulation data, we find that the payoff advantage of cooperators over punishers diminishes as the degree of increasing returns to scale becomes larger. This is because the larger the degree of increasing returns to scale is, the higher the payoff each individual receives from the game. Given that the punishment cost is held fixed, the payoff difference between punishers and cooperators sharply decreases as the average payoff of all individuals increases, indicating that the evolutionary advantage of cooperators over punishers becomes very small (see Supplementary Materials, Fig. S13). When such an advantage becomes sufficiently weak, it is very likely to be offset by the randomness in evolutionary dynamics.

This randomness in the biological evolutionary process is primarily due to genetic variation inside the organism and genetic drift induced by environmental factors. This suggests that biological character is not determined entirely by fitness. Rather, with small probability, it is affected by random disturbances from inside or outside the organisms. If the evolutionary advantage of a particular biological character is large enough, it can successfully resist this random disturbance. Otherwise, its evolutionary advantage will eventually be offset by the random disturbance.

Of course, this randomness affects each type of player. However, our computer simulations show that only punishers, rather than cooperators, can dominate the population. This finding seems to suggest that randomness only weakens the advantage of cooperators. Further analysis of the simulation data shows that cooperators may dominate the population only for transient periods, but eventually they cannot defend their regime because defectors can easily invade and dominate the population.On the contrary, the evolutionary advantage of punishers is reinforced once they become dominant in the population. This is because the abundance of punishers effectively restrains the spread of defectors, reducing the punishment cost. As a result, punishers’ evolutionary advantage becomes even more dominant (see Supplementary Materials, Fig. S14). Therefore, as shown in our computer simulation, once the punishers establish their regime in the population, it becomes extremely difficult for other types to invade.

### The historical evidence of increasing returns to scale

Modern production activities depend not only on labour but also on many other production factors, including capital and technology. However, many large-scale production activities in modern society still depend heavily on the number of cooperators. The number of participants has a great impact on the results of activities. In fact, revenue grows exponentially with the number of participants. Examples of such activities include conventional warfare, geological exploration, and rescue activities during natural calamities such as earthquakes, tsunamis, and floods. As rarely as these events may be considered in modern society, the degrees of increasing returns to scale are usually extremely large in these activities. Our model provides a reasonable interpretation of why individuals are more willing to reach a consensus to cooperate and punish defectors under these circumstances.

In a primitive society in which the level of productivity is extremely low, labour becomes the most important or even the only production factor^1^. The number of contributors thus plays a decisive role in many joint endeavours in a primitive society, such as fighting floods, defending territory, and hunting large game. The degrees of increasing returns to scale are all very large in these activities^52^. Therefore, increasing returns to scale may have been a common feature in most social activities over the prehistorical age, lasting for millions of years.

Ample evolutionary psychology studies have suggested that the mind, and thus the behaviour, of modern man has long been formed by the ancestral environment^53^ because agricultural civilization has a history of only a little more than 10,000 years and industrial civilization is less than 300 years old, whereas human society has millions of years of history. Neuroanatomy evidence also shows that the interconnections among neurons in the human brain have changed very little since the Industrial Revolution. Thus, it may not be surprising that some evolutionary psychologists claim that “our modern skulls house a stone age mind”^54^. These facts help us better understand why the pursuit of fairness and justice has become a common psychological state and the behavioural propensity of human beings. Our results show that natural selection has endowed humankind with a tendency to pursue justice and to punish defectors who deviate from social norms. In other words, the sense of justice is a product of long-lasting human evolution.

## Methods

### A model of a PGG featuring increasing returns to scale

We can apply a Cobb-Douglas production function *P* = *cr*(*X* + *Z*)^*α*^*Y*^*β*^ to characterize the PGG with increasing returns to scale^55^,^56^, where *P* denotes total payoff from the PGG, *c* is the contribution cost to the joint project from each contributor (including cooperators and punishers, *c* > 0), *r* the multiplier of return (*r* > 1), *X* the number of cooperators, *Z* is the number of punishers, *Y* is the number of defectors, and *α* and *β* are the contribution rate of contributors and defectors, respectively. Because *cr* > 0, the PGG is featured with increasing returns to scale if *α* + *β* > 1. Moreover, because defectors make no contribution, namely, *β* = 0, the PGG features increasing returns to scale provided that *α* > 1. In this case, *α* is called the coefficient of increasing returns to scale.

Let *δ* and *γ* denote the strength and the cost of punishment, respectively. Then, the payoffs of cooperators, defectors, and punishers from each period, namely, *P*_*x*_, *P*_*y*_ and *P*_*z,*_from a PGG with increasing returns to scale are given below:


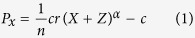



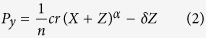



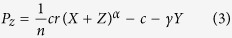


### A stochastic evolutionary model of a PGG with finite population

As a useful method to analyse the stochastic evolutionary process of a finite population, the Moran process is widely applied in studies of biological evolution and evolutionary game theory, e.g., genetic replication, genetic mutation, genetic drift, or strategy learning and updating^57^,^58^,^59^,^60^.

The analysis of the stochastic evolution of a finite population will be greatly simplified in the limiting case, the mutation rate *μ* → 0, where the population consists of two types at most. For *μ* = 0, any monomorphic state becomes absorbing. If the mutation rate *μ* is sufficiently small, a mutant either becomes extinct or spreads into fixation before the next mutant appears. Therefore, the transition between any two monomorphic states occurs only when a mutant appears and spreads into fixation. In this case, the multivariate hypergeometric sampling reduces to a hypergeometric distribution^44^.

Next, consider a sampling process of randomly choosing *N* individuals from a well-mixed finite population of constant size *M* to participate in a PGG with increasing returns to scale. For *μ* → 0, this process is equivalent to an *N*-trial sampling without replacement from a population with *m*_*i*_ individuals of type *i* and *m*_*j*_ = *M*-*m*_*i*_ individuals of type *j*. The probability of selecting *k* individuals of type *i* and *N*-*k* individuals of type *j* is


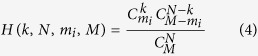


Thus, according to equation (4) and the model of the PGG (1)-(3), in any period of the game, for the *X* cooperators and *Y* = *M*-*X* defectors, the expected payoff *P*_*xy*_ of cooperators competing against defectors and the expected payoff *P*_*yx*_ of defectors competing against cooperators are


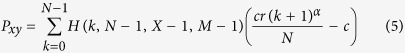



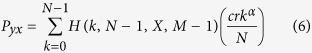


Similarly, the expected payoffs to punishers and defectors are


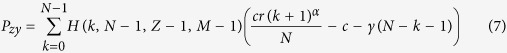



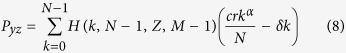


Finally, the expected payoffs to cooperators and punishers are


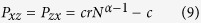


The evolutionary fitness *f*_*ij*_ of an individual of type *i* in a well-mixed population of types *i* and *j* can be calculated from its payoff *P*_*ij*_ and the fitness function *F* = exp(*ωP*), which is


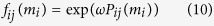


Thus, the probability of changing the number of individuals of type *i* by ±1, *T*_*ij*_^±^, can be calculated


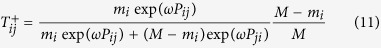



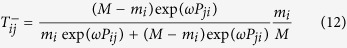


from these transition probabilities, the fixation probability *ρ*_*ij*_ of a single mutant strategy of type *i* in a resident population of type *j* can be derived (see Appendix A):


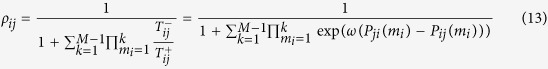


Consequently, the fixation probabilities *ρ*_*ij*_ define the transition probabilities between the three different homogeneous states of the population. The corresponding Markov transition matrix **A** is given by


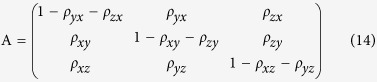


The normalized right eigenvector to the largest eigenvalue (which is 1) of the transposed matrix of **A** determines the stationary distribution; that is, it indicates the probability of finding the system in one of the three homogeneous states. It is given by (see Appendix B)


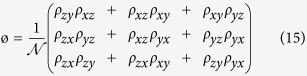


The normalization factor N must be chosen such that the elements of ø sum up to one.

### Simulation

When *μ* > 0 and is not negligible, the fixation probabilities generally continue to fluctuate because of the existence of random disturbance. However, as long as some behaviour or character is evolutionarily stable, its regime in the population will eventually withstand any random disturbance. Here, we adopt a frequency-dependent Moran process to specify the stochastic dynamics of PGG in a finite population^44^,^45^,^47^ and run a series of multi-agent computer simulations with *μ* > 0. The computer simulation procedures are specified as below.

1. Setting a random sample to participate in the game. Applying the Monte Carlo method^60^,^61^,^62^, *N* individuals are randomly chosen from a well-mixed finite population of constant size *M* to participate in a PGG with increasing returns to scale.
2. Calculating the payoffs from the game. Let the individuals from the sample play the game, and then use computer simulation software to calculate the payoff of each type of player at the end of each period of the game according to equations (1–3).
3. Calculating evolutionary fitness. A basic assumption of evolutionary game theory is that individuals are more prone to imitate those with higher payoffs. This assumption implies that individuals with higher payoffs generally have higher fitness and are thus more evolutionarily advantageous. In evolutionary dynamics, a commonly used algorithm to calculate fitness is *F* = 1 − *ω* + *ωP*, where *F* denotes fitness, *P* is payoff, and *ω* is selection strength (0 ＜ *ω* ≤ 1). This algorithm treats fitness as a convex combination of the “baseline fitness”, which is normalized to 1 for all players, and the payoff from the game^44^. A drawback of this algorithm is that it is only applicable for analysing stochastic evolutionary dynamics under weak selection because fitness may be negative for strong selection. To avoid this limitation, we adopt an exponential function *F* = exp(*ωP*) by Thaulsen *et al.*^63^ in our computer simulation, which allows us to accommodate any value of *ω* in its domain.
4. Genetic replication or strategy updating. The Moran process assumes that one member of the population *M* is chosen to die and is replaced by a newly born individual in each generation of the evolutionary process. The type of the newly born individual is jointly determined by both the fitness and frequency of each type of individuals in the population. Usually, two algorithms are commonly used to implement the above process^44^,^59^. The first is the “birth-death” process in which an individual is first chosen for reproduction with a probability proportional to its fitness, and then its clonal offspring replaces a randomly selected individual from the population *M*. The second is the “death-birth” process, in which a randomly selected individual is first removed from the population *M* and another individual is subsequently selected for reproduction with a probability proportional to its fitness and produces a clonal offspring. In addition to these algorithms, we apply a third approach called “genetic pool” in our computer simulations, in which each individual in the population *M* reproduces an offspring with a probability proportional to its fitness, and these newly born individuals form a “genetic pool” from which one offspring is chosen randomly to replace an individual in the population^47^,^59^.
5. Genetic variation or mutation. Genetic variation is an important factor that affects the evolutionary process. A common assumption of evolutionary dynamics is that any individual of a specific type can switch to another type with a small probability *μ* irrespective of its payoff. The parameter *μ* is called the mutation rate. This assumption implies that players will change their strategies with a very small probability without taking the potential payoffs of alternative strategies into account, which can simply be viewed as players’ tentative exploration of alternative strategies^44^.

The aforementioned steps are executed successively and compose our multi-agent computer simulations based on the frequency-dependent Moran process (see Supplementary Materials, Fig. S1).

## Appendix A: Proof of equation (13)

Let *n* denote the state that has *n* individuals of type *i*. From *n* = 0 to *n* = *M*, there are *M* + 1 kinds of states in total, the transitions between which compose the Markov process. The states of *n* = 0 and *n* = *M* are absorbing states. The corresponding Markov transition matrix is


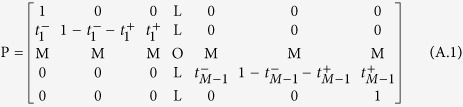


Suppose *x*_*k*_ is the probability of the state *k* transiting to the absorbing state *M*; thus, we can obtain


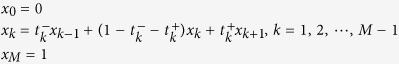


So, if we let *y*_*k*_ = *x*_*k*_ − *x*_*k*−1_, *θ*_*k*_ = *t*_*k*_^−^/*t*_*k*_^+^, we can obtain


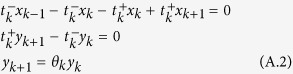


This means that *y*_1_ = *x*_1_, *y*_2_ = *θ*_1_ _×_ _1_, *y*_3_ = *θ*_1_*θ*_2_ _×_ _1_, and so on. We can also obtain the following identity:


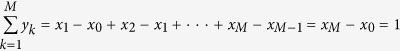


Substituting equation (A.2) into the identity, we can obtain


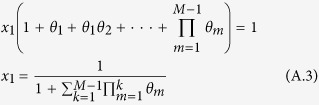


Here, *x*_1_ donates the probability of the state that has a mutant of type *i* transiting to the state completely dominated by this type, which is exactly the fixation probability *ρ*_*ij*_ we want. Substitute *θ* and equation (10) into equation (A.3), and we can obtain equation (13).

## Appendix B: Proof of equation (15)

For a Markov transition matrix **P** = (*P*_*ij*_), a probability distribution {*π*_*i*_, *i* ≥ 0} is called the stationary distribution of the Markov chain if it satisfies


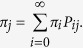


It can be rewritten as


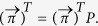


Taking the transpose of both sides, we can obtain


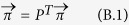


The transposed matrix of the Markov transition matrix **A** is


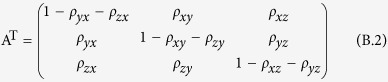


Substituting equation (B.2) into equation (B.1) and with some calculations, we can obtain the following homogeneous linear equations:


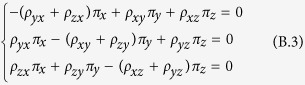


Remember that {*π*_*i*_, *i* ≥ 0} is a probability distribution, which means the identity


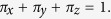


Substituting the identity into equation (B.3), we can obtain equation (15).

## Additional Information

**How to cite this article**: Ye, H. *et al.* Increasing returns to scale: The solution to the second-order social dilemma. *Sci. Rep.* **6**, 31927; doi: 10.1038/srep31927 (2016).

## Supplementary Material

Supplementary Information

## Figures and Tables

**Figure 1:**
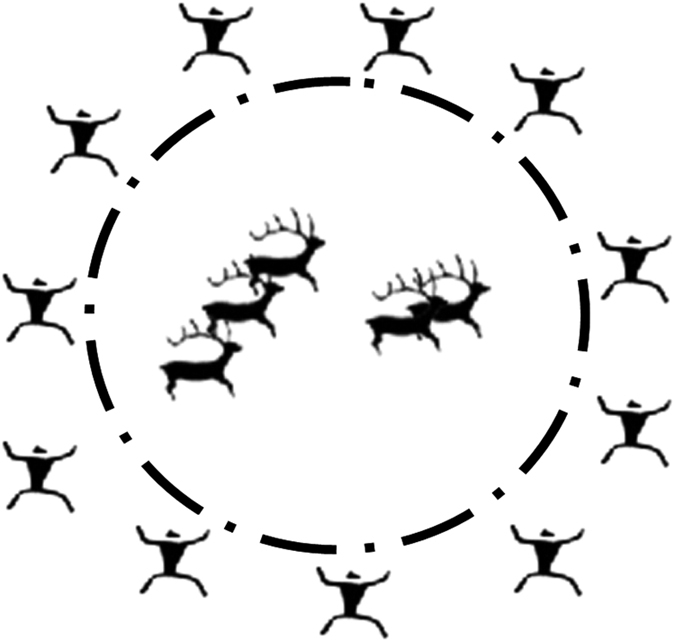
Collective hunting in a primitive society.
