# Supplementary material for: Increasing returns to scale: The solution to the second-order social dilemma
Source: Sci Rep. 2016 Aug 18;6:31927. doi: 10.1038/srep31927 (PMC4989174; doi:10.1038/srep31927)
Supplement: Supplementary Information [file srep31927-s1.pdf]

Supplementary Materials

Scientific Reports

Increasing returns to scale: The solution to the second-order social dilemma

Hang Ye, Shu Chen, Jun Luo, Fei Tan, Yongmin Jia and Yefeng Chen

Author for correspondence: Yefeng Chen
College of Economics and Interdisciplinary Center for Social Sciences, Zhejiang University, No 38, Zheda Road, Hangzhou 310027, China
E-mail: lenggone@gmail.com

Table of Contents

1 The computer simulation process p. 1-1
2 Robust tests p. 2-11
3 Figures of the simulation data p. 12-13
4 Analysis of the simulation data p. 14-26

1 The computer simulation process

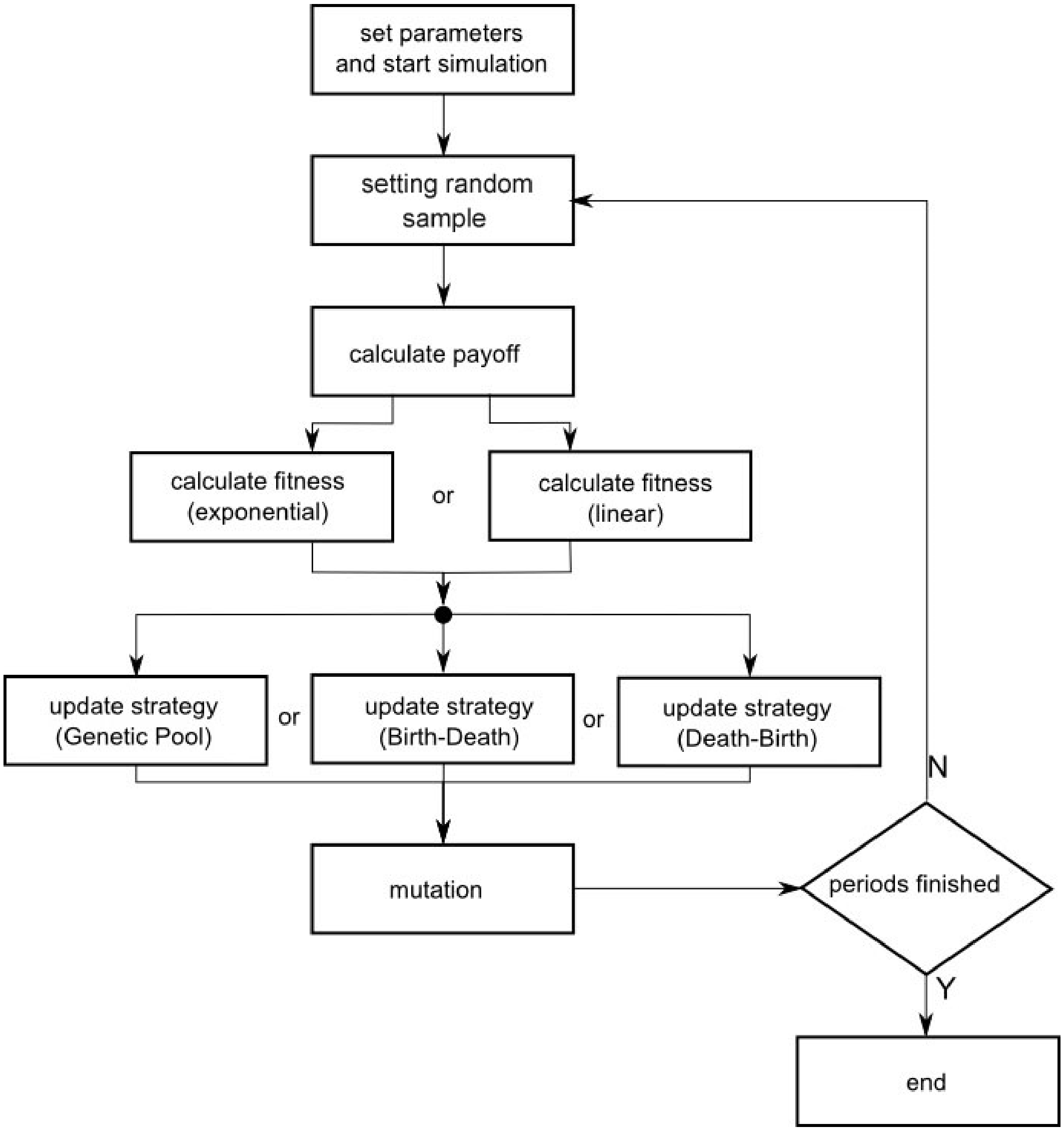

Figure S1. Simulation process

We offer supporting online simulation, which can be downloaded and run via the Internet: http://mypage.zju.edu.cn/en/yehang/631163.html.

2 The Robust Tests of Computer Simulation

2.1 Periods

In computer simulation with periods over millions, can punishers still be dominating the population? We conducted the simulations with $\text{periods} = 10^6$ and $\text{periods} = 10^7$. It turned out that punishers are significantly more evolutionarily advantageous against defectors if the degrees of increasing returns to scale are large enough. Parameter values are $M = 100$, $N = 5$, $X = 30$, $Y = 40$, $Z = 30$, $c = 1$, $r = 3$, $\delta = 1$, $\gamma = 0.3$, $\omega = 0.5$, $\mu = 0.001$, and $\alpha = 1.0$ (in Figure 2A and 3A) or $\alpha = 1.8$ (in Figure 2B and 3B). Fitness function: exponential function. Moran process: genetic pool.

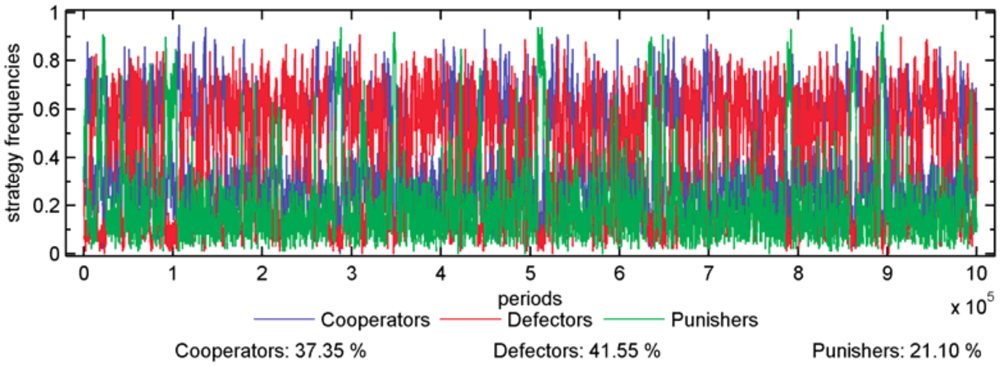

Figure S2A. Simulation result of public goods game with periods=10^6 and constant returns to scale.

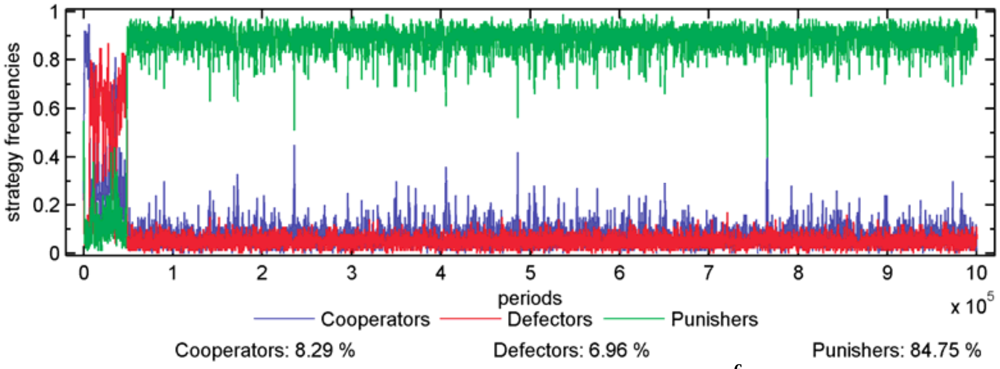

Figure S2B. Simulation result of public goods game with periods=10^6 and increasing returns to scale.

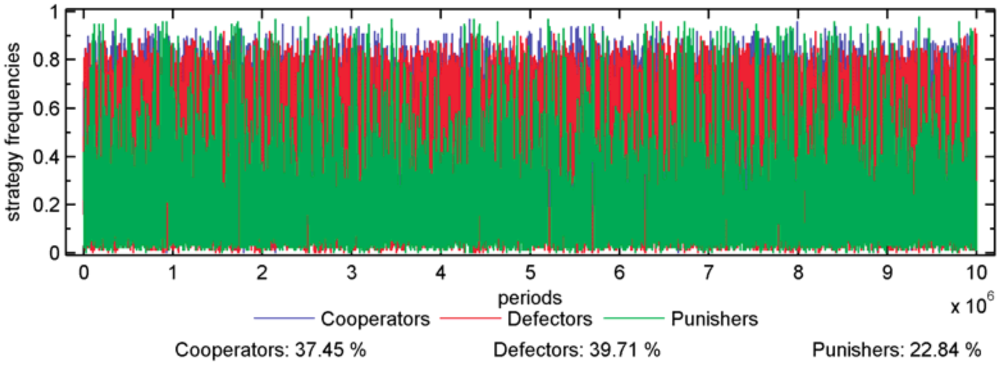

Figure S3A. Simulation result of public goods game with periods=10^7 and constant returns to scale.

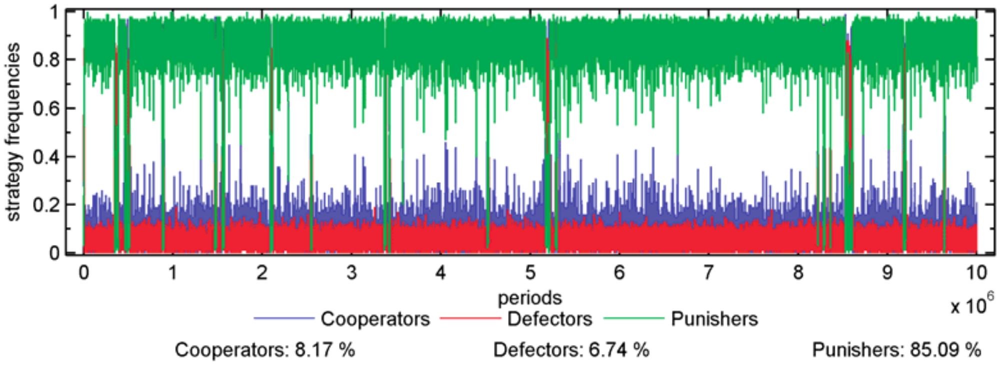

Figure S3B. Simulation result of public goods game with periods=10^7 and increasing returns to scale.

2.2 Initial population

In different initial population, can the simulation results maintain stable? We conducted the simulations with initial population consists only of defectors, cooperators, or punishers. It turned out that the results hold firm and still. Parameter values are periods=10^5, $M=100$, $N=5$, $c=1$, $r=3$, $\delta=1$, $\gamma=0.3$, $\omega=0.5$, $\mu=0.001$, and $\alpha=1.0$ (in Figure S4A, S5A, and S6A) or $\alpha=1.8$ (in Figure S4B, S5B, and S6B). Fitness function: exponential function. Moran process: genetic pool.

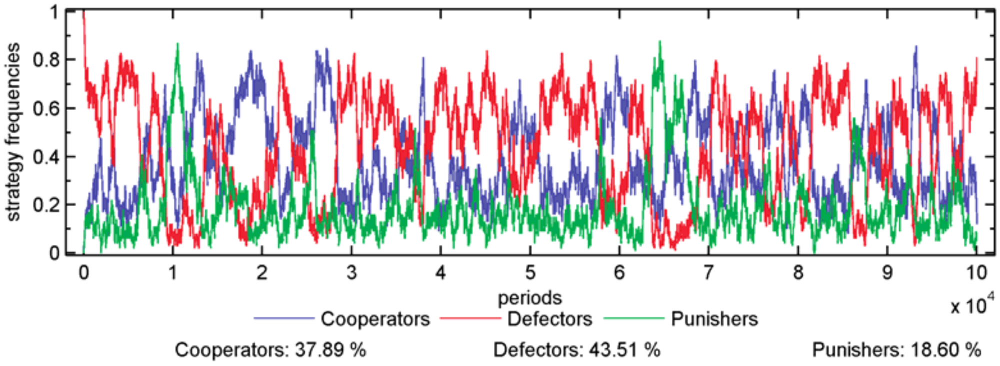

Figure S4A. The public goods game of constant returns to scale with initial population consists only of defectors.

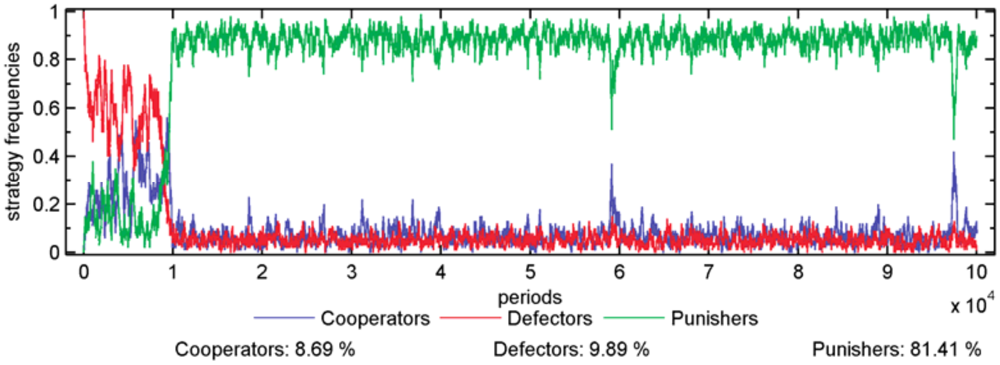

Figure S4B. The public goods game of increasing returns to scale with initial population consists only of defectors.

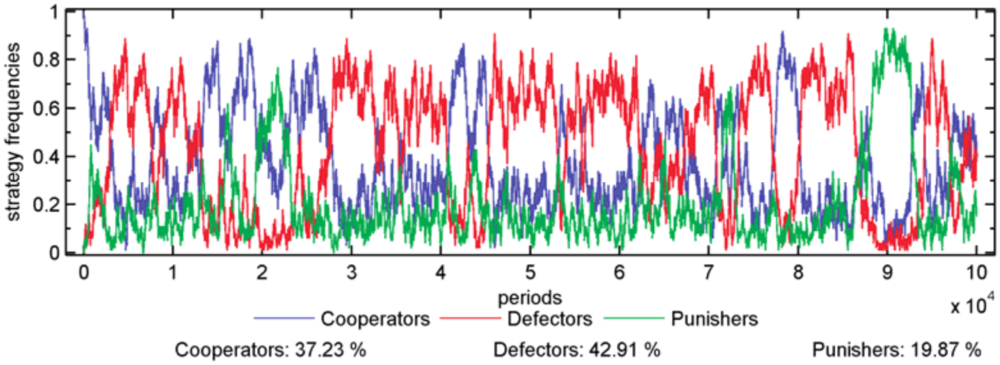

Figure S5A. The public goods game of constant returns to scale with initial population consists only of cooperators.

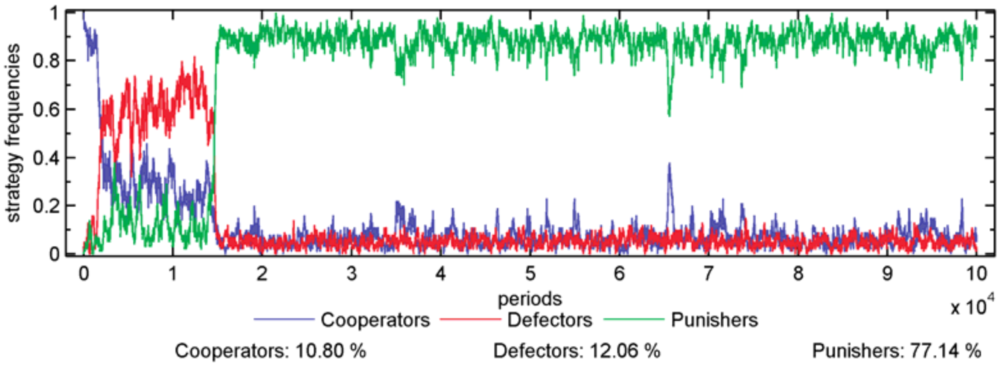

Figure S5B. The public goods game of increasing returns to scale with initial population consists only of cooperators.

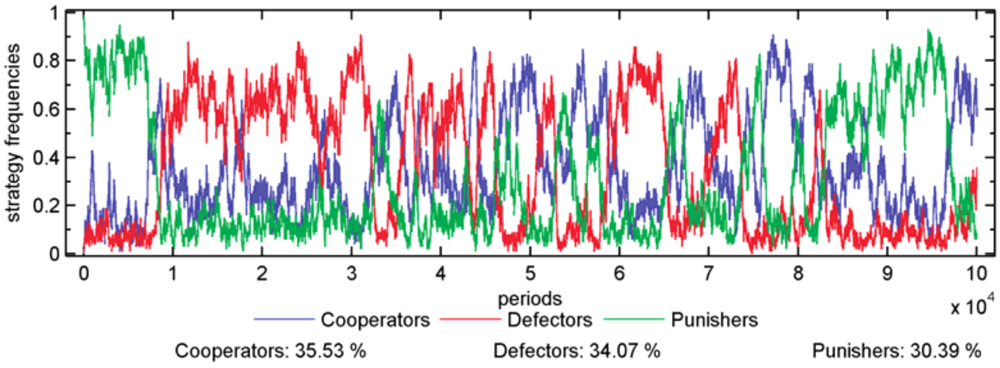

Figure S6A. The public goods game of constant returns to scale with initial population consists only of punishers.

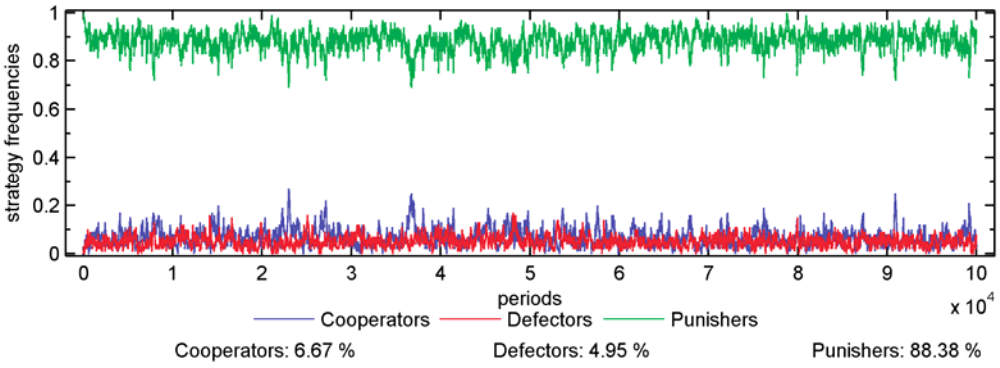

Figure S6B. The public goods game of increasing returns to scale with initial population consists only of punishers.

2.3 The contribution rate of contributors (α)

Do different levels of economy of scale affect the stability of our results? We conducted the simulations with $\alpha = 1.0 \sim 2.0$ (with step length=0.2). The results showed that, with α increasing, the punishers become more evolutionarily advantageous. Parameter values are periods=10^5, $M = 100$, $N = 5$, $X = 30$, $Y = 40$, $Z = 30$, $c = 1$, $r = 3$, $\delta = 1$, $\gamma = 0.3$, $\omega = 0.5$, $\mu = 0.001$. Fitness function: exponential function. Moran process: genetic pool.

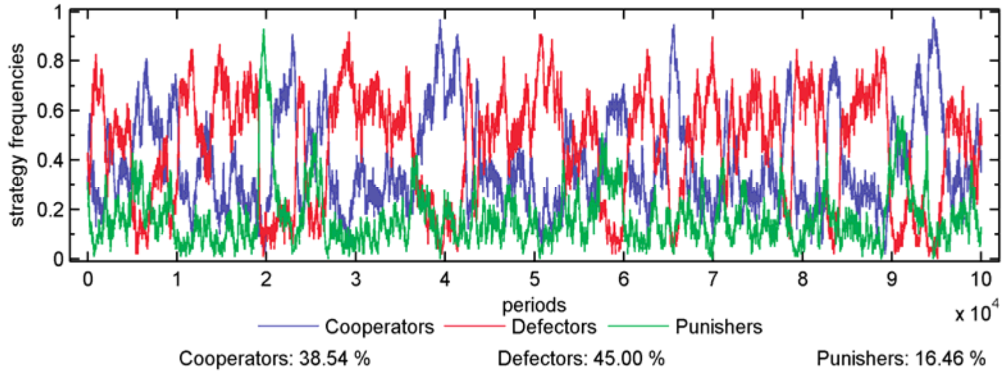

Figure S7A. The public goods game with $\alpha = 1.0$.

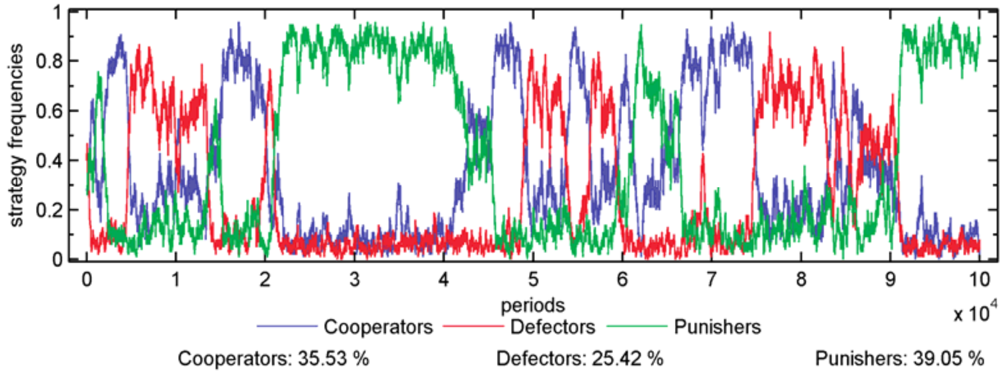

Figure S7B. The public goods game with $\alpha = 1.2$.

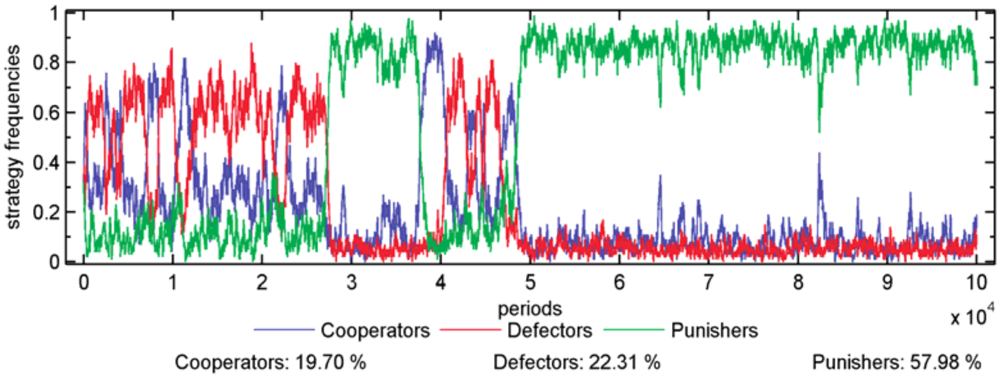

Figure S7C. The public goods game with $\alpha = 1.4$ **.**

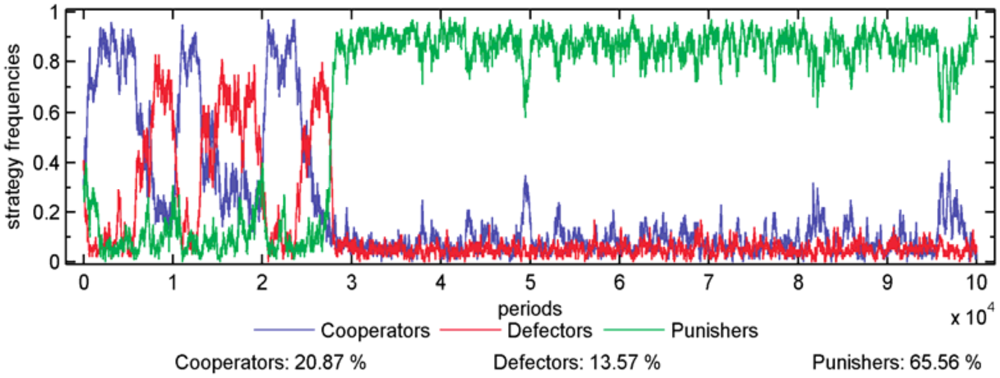

Figure S7D. The public goods game with $\alpha = 1.6$ **.**

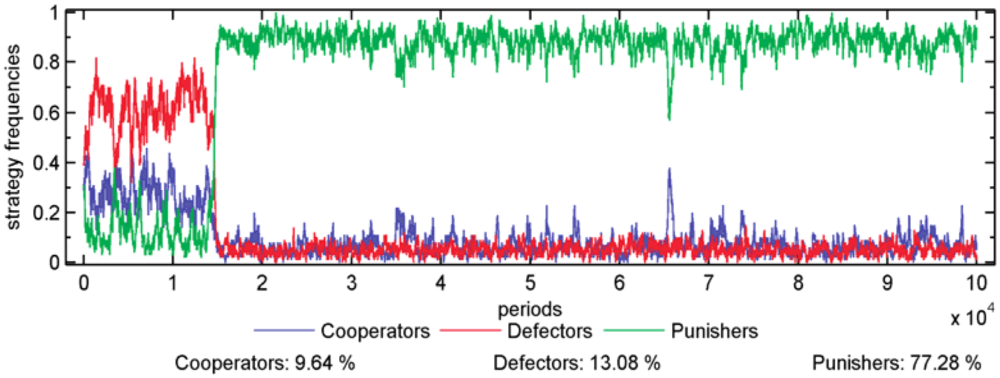

Figure S7E. The public goods game with $\alpha = 1.8$ **.**

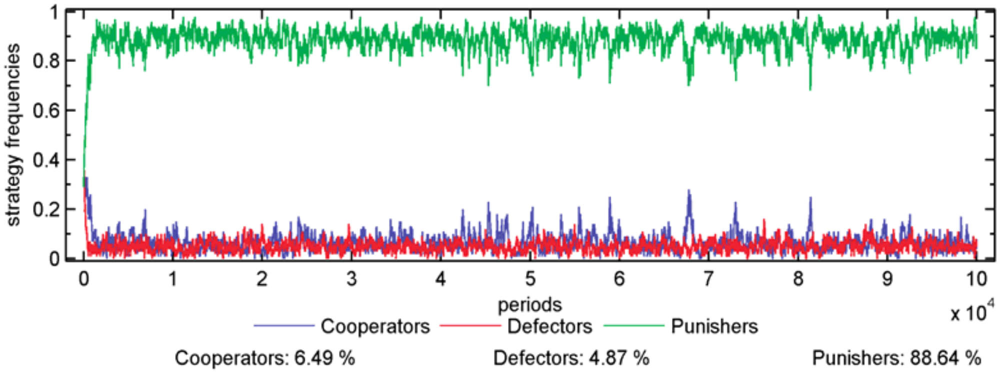

Figure S7F. The public goods game with $\alpha = 2.0$.

2.4 The fitness functions

Do our results withstand alternative fitness functions? We conducted the simulations with linear fitness function and exponential fitness function. The results confirmed our conclusions. Parameter values are periods=10^5, $M = 100$, $N = 5$, $X = 30$, $Y = 40$, $Z = 30$, $c = 1$, $r = 3$, $\delta = 1$, $\gamma = 0.3$, $\mu = 0.001$, $\omega = 0.3$, and $\alpha = 1.0$ (in Figure S8A and S9A) or $\alpha = 1.8$ (in Figure S8B and S9B). Moran process: genetic pool.

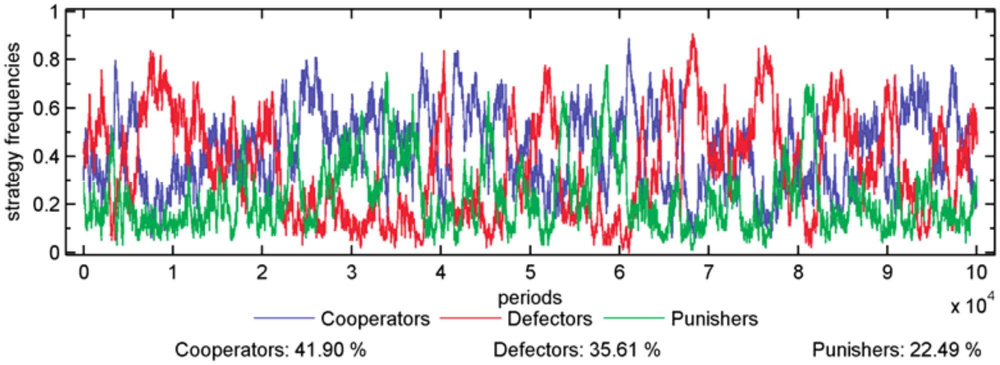

Figure S8A. The public goods game with linear fitness function and $\alpha = 1.0$.

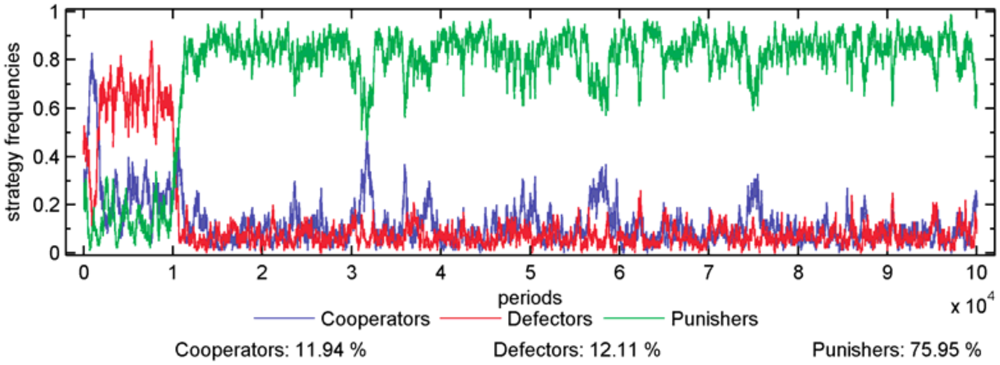

Figure S8B. The public goods game with linear fitness function and $\alpha = 1.8$.

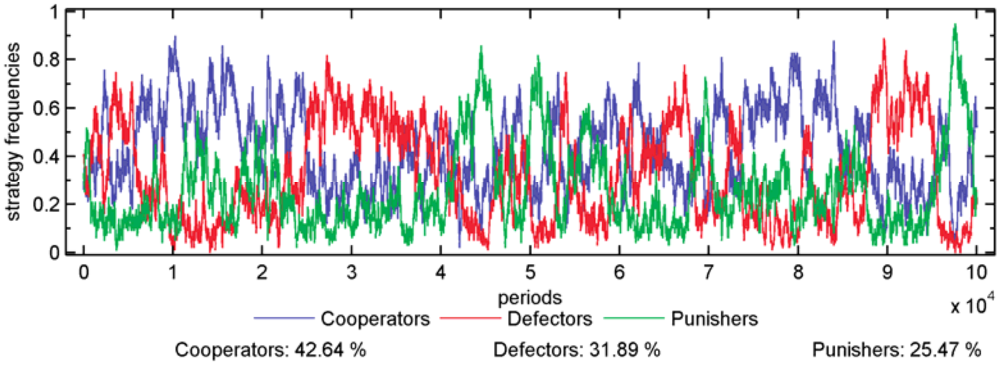

Figure S9A. The public goods game with exponential fitness function and $\alpha = 1.0$ **.**

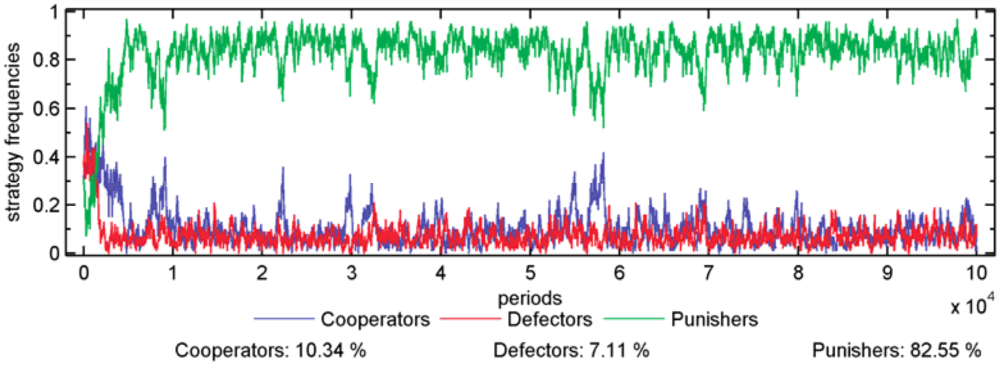

Figure S9B. The public goods game with exponential fitness function and $\alpha = 1.8$ **.**

2.6 The Moran processes

Do our results differ with the specified Moran processes? We conducted the simulations with the Moran processes of death-birth, birth-death and genetic pool. Our results stay stable. Parameter values are periods=10^5, $M = 100$, $N = 5$, $X = 30$, $Y = 40$, $Z = 30$, $c = 1$, $r = 3$, $\delta = 1$, $\gamma = 0.3$, $\mu = 0.001$, $\omega = 0.5$, and $\alpha = 1.0$ (in Figure S10A, S11A, and S12A) or $\alpha = 1.8$ (in Figure S10B, S11B, and S12B). Fitness function: exponential function.

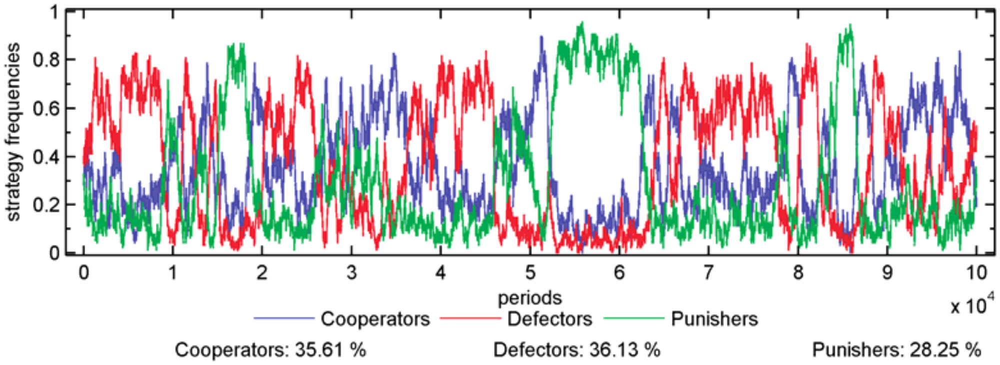

Figure S10A. The public goods game with the Moran process of death-birth and $\alpha = 1.0$.

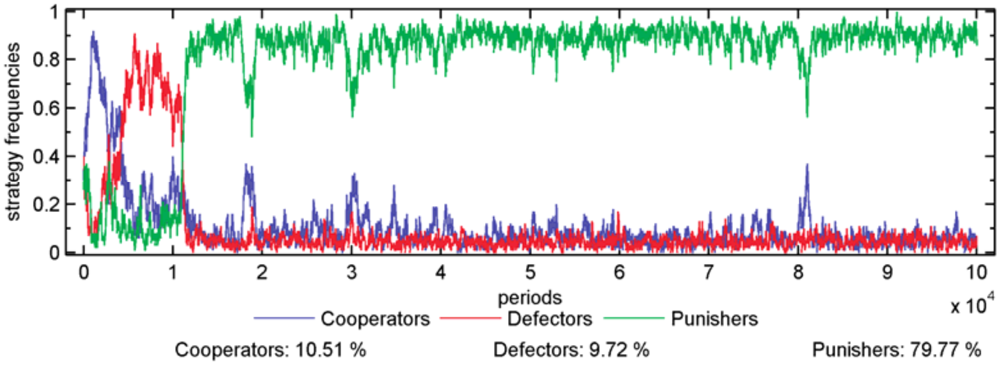

Figure S10B. The public goods game with the Moran process of death-birth and $\alpha = 1.8$.

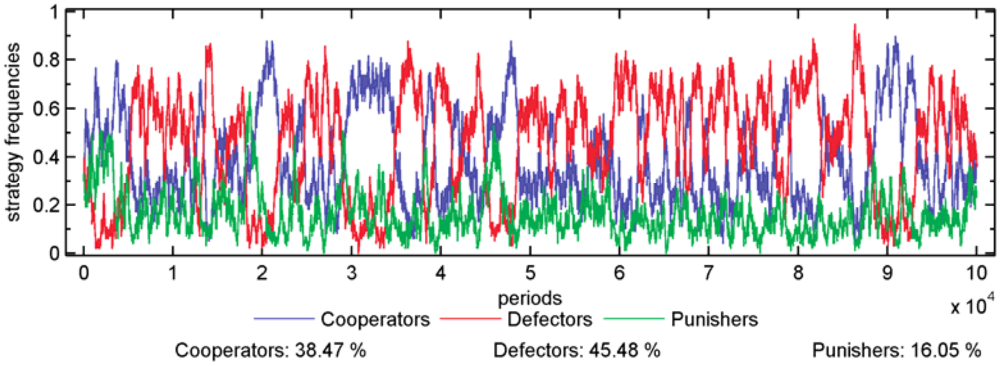

Figure S11A. The public goods game with the Moran process of birth-death and $\alpha = 1.0$.

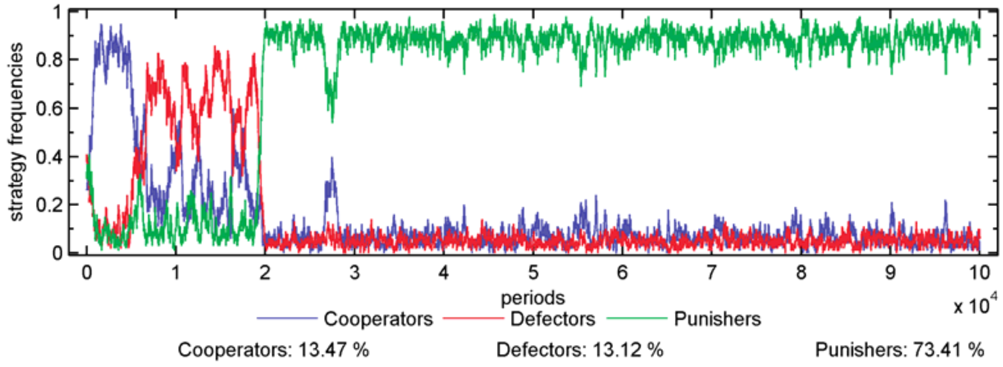

Figure S11B. The public goods game with the Moran process of birth-death and $\alpha = 1.8$.

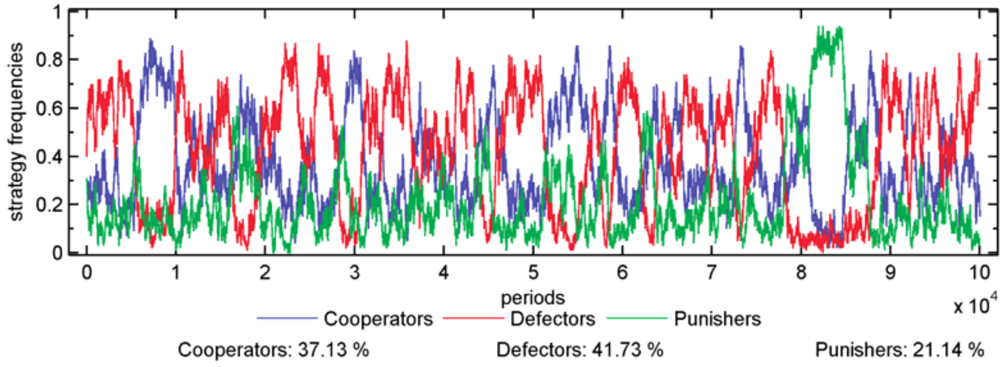

Figure S12A. The public goods game with the Moran process of genetic pool and $\alpha = 1.0$.

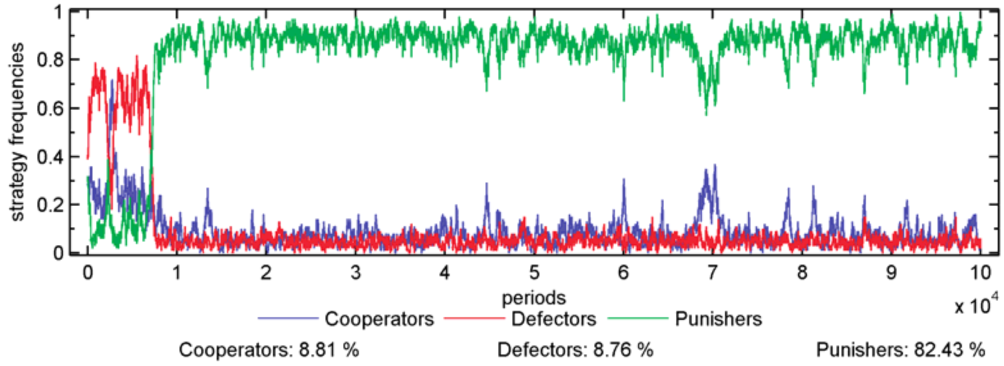

Figure S12B. The public goods game with the Moran process of genetic pool and $\alpha = 1.8$.

More robust tests are available online in our website：
http://mypage.zju.edu.cn/en/yehang/631144.html.

3 Figures of the Simulation Data

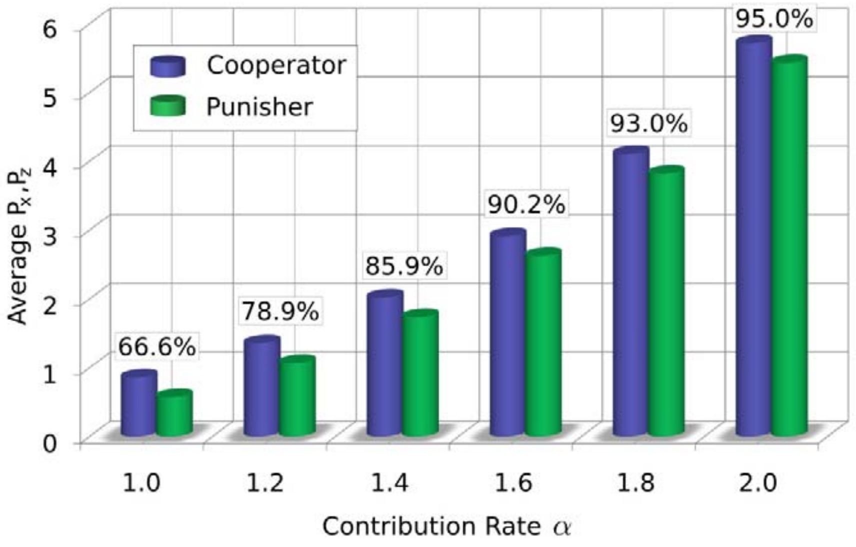

Figure S13. Payoff comparisons between cooperators and punishers with different α values. Parameter values are M=100, N=5 (N_x=2; N_y=1; N_z=2), X=30, Y=40, Z=30, c=1, r=3, α=10~2.0, δ=1, γ=0.3, ω=0.5, μ=0.001. As the returns to scale increase, the payoff difference decreases, which indicates that the payoff advantage of cooperators over punishers is reduced. For more details, see table S3 of analysis to the simulation data in electronic supplementary material.

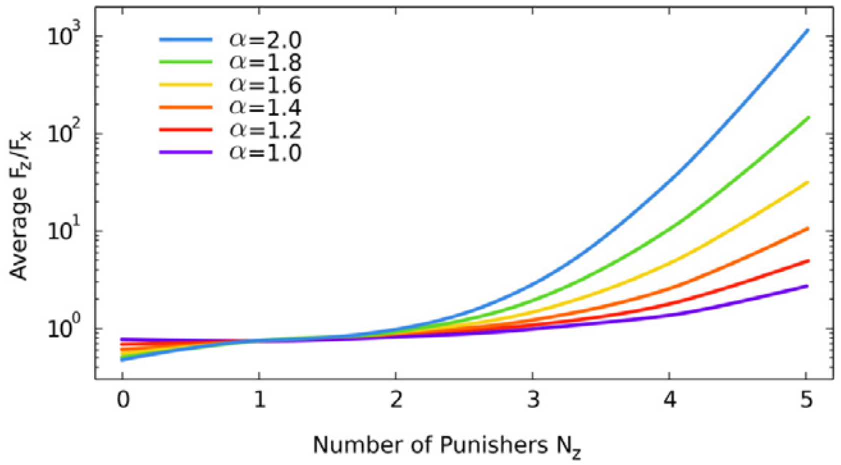

Figure S14. The effect of the number of punishers on fitness. The horizontal axis represents the number of punishers selected (N_z). The vertical axis (logarithm scale) is the ratio of the average fitness of punishers and cooperators in N (F_z/F_x), which indicates the relative advantage the punishers have over cooperators with regard to fitness. As the figure shows, the relative fitness advantage (F_z/F_x) has a positive feedback relation with the number of punishers (N_z). F_z/F_x increases with increasing numbers of punishers, and this becomes more obvious with the increasing economy of scale (α). For more details, see table S5 of analysis to the simulation data in electronic supplementary material.

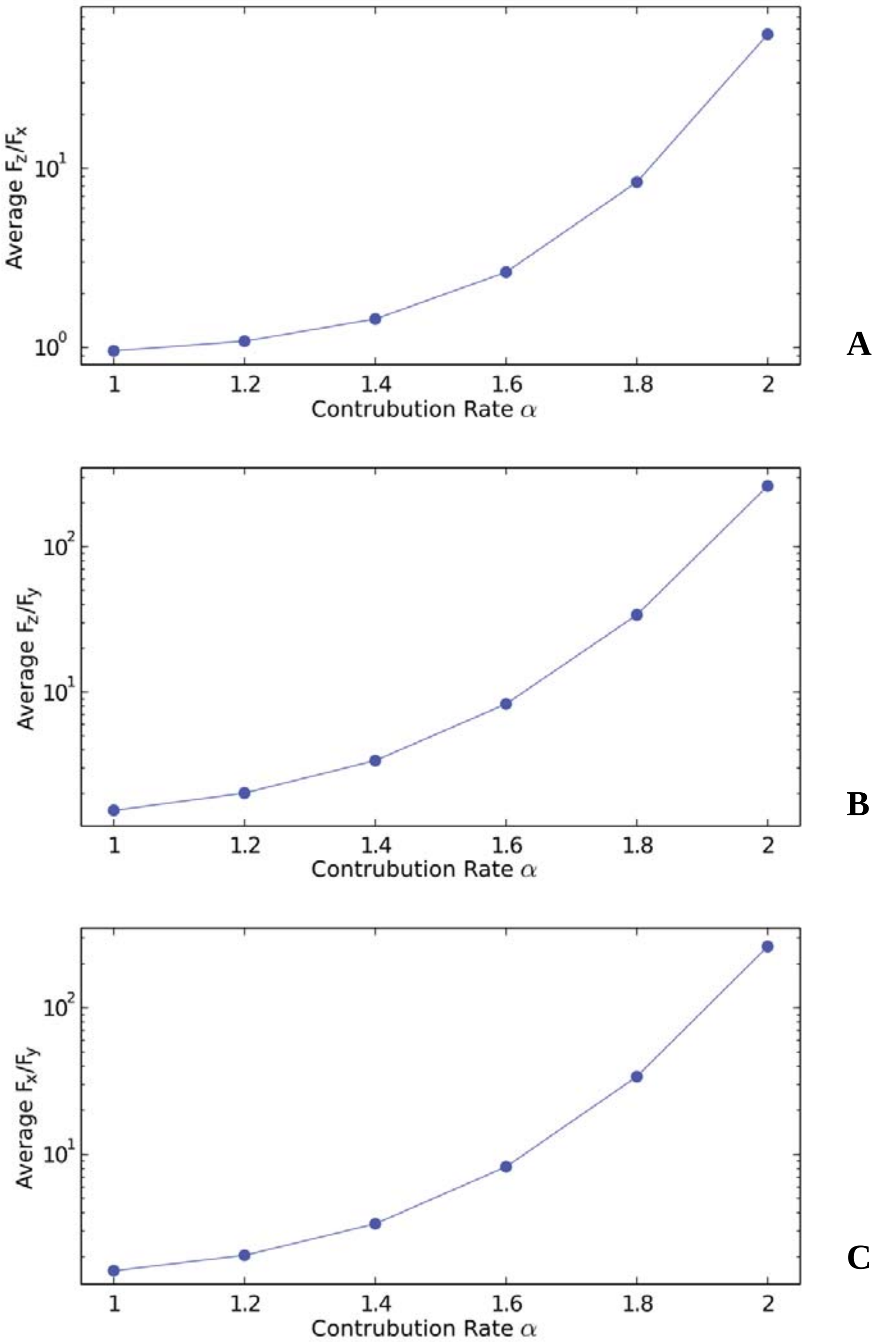

Figure S15. The relative fitness of punishers to cooperators (A), **punishers to defectors (B), and cooperators to defectors (C) with** $\alpha = 1.0 \sim 2.0$. The figure is based on the data in Table S2 of analysis to the simulation data in electronic supplementary material. All the three pictures clearly show that, both punishers and cooperators are significantly more evolutionarily advantageous as α increases.

4 Analysis to the Simulation Data

The following 3 tables present the original data from the computer simulation. When $N = 5$, there are 21 combinations of the three types of the individuals in N. Table S1is the payoffs of the three types of the individuals in N with $\alpha = 1.0 \sim 2.0$; Table S2 is the fitnesses of the three types of the individuals in N with $\alpha = 1.0 \sim 2.0$; Table S3 is the relative payoffs of punishers to cooperators (**A**), cooperators to defectors (**B**), and punishers to defectors (**C**) with $\alpha = 1.0 \sim 2.0$ **;** Table S4 is the relative fitnesses of punishers to cooperators (**A**), cooperators to defectors (**B**), and punishers to defectors (**C**) with $\alpha = 1.0 \sim 2.0$ **;** Table S5 is the average relative fitnesses of punishers to cooperators with different punishers amount in N and $\alpha = 1.0 \sim 2.0$.

Table S1A. The payoffs of the three types of the individuals in N with $\alpha = 1.0$ and $\alpha = 1.2$.

N_x	N_y	N_z	P_x ($\alpha = 1.0$)	P_y ($\alpha = 1.0$)	P_z ($\alpha = 1.0$)	P_x ($\alpha = 1.2$)	P_y ($\alpha = 1.2$)	P_z ($\alpha = 1.2$)
**0**	**0**	**5**	0.000000	0.000000	2.000000	0.000000	0.000000	3.139189
**0**	**1**	**4**	0.000000	-1.600000	1.100000	0.000000	-0.833181	1.866819
**0**	**2**	**3**	0.000000	-1.200000	0.200000	0.000000	-0.757684	0.642316
**0**	**3**	**2**	0.000000	-0.800000	-0.700000	0.000000	-0.621562	-0.521562
**0**	**4**	**1**	0.000000	-0.400000	-1.600000	0.000000	-0.400000	-1.600000
**0**	**5**	**0**	0.000000	0.000000	0.000000	0.000000	0.000000	0.000000
**1**	**0**	**4**	2.000000	0.000000	2.000000	3.139189	0.000000	3.139189
**1**	**1**	**3**	1.400000	-0.600000	1.100000	2.166819	0.166819	1.866819
**1**	**2**	**2**	0.800000	-0.200000	0.200000	1.242316	0.242316	0.642316
**1**	**3**	**1**	0.200000	0.200000	-0.700000	0.378438	0.378438	-0.521562
**1**	**4**	**0**	-0.400000	0.600000	0.000000	-0.400000	0.600000	0.000000
**2**	**0**	**3**	2.000000	0.000000	2.000000	3.139189	0.000000	3.139189
**2**	**1**	**2**	1.400000	0.400000	1.100000	2.166819	1.166819	1.866819
**2**	**2**	**1**	0.800000	0.800000	0.200000	1.242316	1.242316	0.642316
**2**	**3**	**0**	0.200000	1.200000	0.000000	0.378438	1.378438	0.000000
**3**	**0**	**2**	2.000000	0.000000	2.000000	3.139189	0.000000	3.139189
**3**	**1**	**1**	1.400000	1.400000	1.100000	2.166819	2.166819	1.866819
**3**	**2**	**0**	0.800000	1.800000	0.000000	1.242316	2.242316	0.000000
**4**	**0**	**1**	2.000000	0.000000	2.000000	3.139189	0.000000	3.139189
**4**	**1**	**0**	1.400000	2.400000	0.000000	2.166819	3.166819	0.000000
**5**	**0**	**0**	2.000000	0.000000	0.000000	3.139189	0.000000	0.000000

Table S1B. The payoffs of the three types of the individuals in N with $\alpha = 1.4$ and $\alpha = 1.6$.

N_x	N_y	N_z	P_x ($\alpha = 1.4$)	P_y ($\alpha = 1.4$)	P_z ($\alpha = 1.4$)	P_x ($\alpha = 1.6$)	P_y ($\alpha = 1.6$)	P_z ($\alpha = 1.6$)
**0**	**0**	**5**	0.000000	0.000000	4.710962	0.000000	0.000000	6.879583
**0**	**1**	**4**	0.000000	0.178643	2.878643	0.000000	1.513752	4.213752
**0**	**2**	**3**	0.000000	-0.206678	1.193322	0.000000	0.479728	1.879728
**0**	**3**	**2**	0.000000	-0.416591	-0.316591	0.000000	-0.181140	-0.081140
**0**	**4**	**1**	0.000000	-0.400000	-1.600000	0.000000	-0.400000	-1.600000
**0**	**5**	**0**	0.000000	0.000000	0.000000	0.000000	0.000000	0.000000
**1**	**0**	**4**	4.710962	0.000000	4.710962	6.879583	0.000000	6.879583
**1**	**1**	**3**	3.178643	1.178643	2.878643	4.513752	2.513752	4.213752
**1**	**2**	**2**	1.793322	0.793322	1.193322	2.479728	1.479728	1.879728
**1**	**3**	**1**	0.583409	0.583409	-0.316591	0.818860	0.818860	-0.081140
**1**	**4**	**0**	-0.400000	0.600000	0.000000	-0.400000	0.600000	0.000000
**2**	**0**	**3**	4.710962	0.000000	4.710962	6.879583	0.000000	6.879583
**2**	**1**	**2**	3.178643	2.178643	2.878643	4.513752	3.513752	4.213752
**2**	**2**	**1**	1.793322	1.793322	1.193322	2.479728	2.479728	1.879728
**2**	**3**	**0**	0.583409	1.583409	0.000000	0.818860	1.818860	0.000000
**3**	**0**	**2**	4.710962	0.000000	4.710962	6.879583	0.000000	6.879583
**3**	**1**	**1**	3.178643	3.178643	2.878643	4.513752	4.513752	4.213752
**3**	**2**	**0**	1.793322	2.793322	0.000000	2.479728	3.479728	0.000000
**4**	**0**	**1**	4.710962	0.000000	4.710962	6.879583	0.000000	6.879583
**4**	**1**	**0**	3.178643	4.178643	0.000000	4.513752	5.513752	0.000000
**5**	**0**	**0**	4.710962	0.000000	0.000000	6.879583	0.000000	0.000000

Table S1C. The payoffs of the three types of the individuals in N with $\alpha = 1.8$ and $\alpha = 2.0$.

N_x	N_y	N_z	P_x ($\alpha = 1.8$)	P_y ($\alpha = 1.8$)	P_z ($\alpha = 1.8$)	P_x ($\alpha = 2.0$)	P_y ($\alpha = 2.0$)	P_z ($\alpha = 2.0$)
**0**	**0**	**5**	0.000000	0.000000	9.871695	0.000000	0.000000	14.000000
**0**	**1**	**4**	0.000000	3.275440	5.975440	0.000000	5.600000	8.300000
**0**	**2**	**3**	0.000000	1.334804	2.734804	0.000000	2.400000	3.800000
**0**	**3**	**2**	0.000000	0.089321	0.189321	0.000000	0.400000	0.500000
**0**	**4**	**1**	0.000000	-0.400000	-1.600000	0.000000	-0.400000	-1.600000
**0**	**5**	**0**	0.000000	0.000000	0.000000	0.000000	0.000000	0.000000
**1**	**0**	**4**	9.871695	0.000000	9.871695	14.000000	0.000000	14.000000
**1**	**1**	**3**	6.275440	4.275440	5.975440	8.600000	6.600000	8.300000
**1**	**2**	**2**	3.334804	2.334804	2.734804	4.400000	3.400000	3.800000
**1**	**3**	**1**	1.089321	1.089321	0.189321	4.400000	4.400000	3.800000
**1**	**4**	**0**	-0.400000	0.600000	0.000000	-0.400000	0.600000	0.000000
**2**	**0**	**3**	9.871695	0.000000	9.871695	14.000000	0.000000	14.000000
**2**	**1**	**2**	6.275440	5.275440	5.975440	8.600000	7.600000	8.300000
**2**	**2**	**1**	3.334804	3.334804	2.734804	4.400000	4.400000	3.800000
**2**	**3**	**0**	1.089321	2.089321	0.000000	1.400000	2.400000	0.000000
**3**	**0**	**2**	9.871695	0.000000	9.871695	14.000000	0.000000	14.000000
**3**	**1**	**1**	6.275440	6.275440	5.975440	8.600000	8.600000	8.300000
**3**	**2**	**0**	3.334804	4.334804	0.000000	4.400000	5.400000	0.000000
**4**	**0**	**1**	9.871695	0.000000	9.871695	14.000000	0.000000	14.000000
**4**	**1**	**0**	6.275440	7.275440	0.000000	8.600000	9.600000	0.000000
**5**	**0**	**0**	9.871695	0.000000	0.000000	14.000000	0.000000	0.000000

Table S2A. The fitnesses of the three types of the individuals in N with $\alpha = 1.0$ and $\alpha = 1.2$.

N_x	N_y	N_z	F_x ($\alpha = 1.0$)	F_y ($\alpha = 1.0$)	F_z ($\alpha = 1.0$)	F_x ($\alpha = 1.2$)	F_y ($\alpha = 1.2$)	F_z ($\alpha = 1.2$)
**0**	**0**	**5**	1.000000	1.000000	2.718282	1.000000	1.000000	4.804699
**0**	**1**	**4**	1.000000	0.449329	1.733253	1.000000	0.659291	2.543165
**0**	**2**	**3**	1.000000	0.548812	1.105171	1.000000	0.684654	1.378723
**0**	**3**	**2**	1.000000	0.670320	0.704688	1.000000	0.732874	0.770450
**0**	**4**	**1**	1.000000	0.818731	0.449329	1.000000	0.818731	0.449329
**0**	**5**	**0**	1.000000	1.000000	1.000000	1.000000	1.000000	1.000000
**1**	**0**	**4**	2.718282	1.000000	2.718282	4.804699	1.000000	4.804699
**1**	**1**	**3**	2.013753	0.740818	1.733253	2.954737	1.086987	2.543165
**1**	**2**	**2**	1.491825	0.904837	1.105171	1.861082	1.128803	1.378723
**1**	**3**	**1**	1.105171	1.105171	0.704688	1.208306	1.208306	0.770450
**1**	**4**	**0**	0.818731	1.349859	1.000000	0.818731	1.349859	1.000000
**2**	**0**	**3**	2.718282	1.000000	2.718282	4.804699	1.000000	4.804699
**2**	**1**	**2**	2.013753	1.221403	1.733253	2.954737	1.792138	2.543165
**2**	**2**	**1**	1.491825	1.491825	1.105171	1.861082	1.861082	1.378723
**2**	**3**	**0**	1.105171	1.822119	1.000000	1.208306	1.992159	1.000000
**3**	**0**	**2**	2.718282	1.000000	2.718282	4.804699	1.000000	4.804699
**3**	**1**	**1**	2.013753	2.013753	1.733253	2.954737	2.954737	2.543165
**3**	**2**	**0**	1.491825	2.459603	1.000000	1.861082	3.068405	1.000000
**4**	**0**	**1**	2.718282	1.000000	2.718282	4.804699	1.000000	4.804699
**4**	**1**	**0**	2.013753	3.320117	1.000000	2.954737	4.871537	1.000000
**5**	**0**	**0**	2.718282	1.000000	1.000000	4.804699	1.000000	1.000000

Table S2B. The fitnesses of the three types of the individuals in N with $\alpha = 1.4$ and $\alpha = 1.6$.

N_x	N_y	N_z	F_x ($\alpha = 1.4$)	F_y ($\alpha = 1.4$)	F_z ($\alpha = 1.4$)	F_x ($\alpha = 1.6$)	F_y ($\alpha = 1.6$)	F_z ($\alpha = 1.6$)
**0**	**0**	**5**	1.000000	1.000000	10.543198	1.000000	1.000000	31.180463
**0**	**1**	**4**	1.000000	1.093432	4.217832	1.000000	2.131607	8.222514
**0**	**2**	**3**	1.000000	0.901821	1.816045	1.000000	1.271076	2.559633
**0**	**3**	**2**	1.000000	0.811967	0.853598	1.000000	0.913410	0.960242
**0**	**4**	**1**	1.000000	0.818731	0.449329	1.000000	0.818731	0.449329
**0**	**5**	**0**	1.000000	1.000000	1.000000	1.000000	1.000000	1.000000
**1**	**0**	**4**	10.543198	1.000000	10.543198	31.180463	1.000000	31.180463
**1**	**1**	**3**	4.900422	1.802765	4.217832	9.553199	3.514425	8.222514
**1**	**2**	**2**	2.451404	1.486852	1.816045	3.455143	2.095650	2.559633
**1**	**3**	**1**	1.338708	1.338708	0.853598	1.505959	1.505959	0.960242
**1**	**4**	**0**	0.818731	1.349859	1.000000	0.818731	1.349859	1.000000
**2**	**0**	**3**	10.543198	1.000000	10.543198	31.180463	1.000000	31.180463
**2**	**1**	**2**	4.900422	2.972256	4.217832	9.553199	5.794308	8.222514
**2**	**2**	**1**	2.451404	2.451404	1.816045	3.455143	3.455143	2.559633
**2**	**3**	**0**	1.338708	2.207156	1.000000	1.505959	2.482907	1.000000
**3**	**0**	**2**	10.543198	1.000000	10.543198	31.180463	1.000000	31.180463
**3**	**1**	**1**	4.900422	4.900422	4.217832	9.553199	9.553199	8.222514
**3**	**2**	**0**	2.451404	4.041682	1.000000	3.455143	5.696568	1.000000
**4**	**0**	**1**	10.543198	1.000000	10.543198	31.180463	1.000000	31.180463
**4**	**1**	**0**	4.900422	8.079430	1.000000	9.553199	15.750562	1.000000
**5**	**0**	**0**	10.543198	1.000000	1.000000	31.180463	1.000000	1.000000

Table S2C. The fitnesses of the three types of the individuals in N with $\alpha = 1.8$ and $\alpha = 2.0$.

N_x	N_y	N_z	F_x ($\alpha = 1.4$)	F_y ($\alpha = 1.4$)	F_z ($\alpha = 1.4$)	F_x ($\alpha = 1.6$)	F_y ($\alpha = 1.6$)	F_z ($\alpha = 1.6$)
**0**	**0**	**5**	1.000000	1.000000	139.191054	1.000000	1.000000	1096.633163
**0**	**1**	**4**	1.000000	5.143428	19.840390	1.000000	16.444647	63.434000
**0**	**2**	**3**	1.000000	1.949167	3.925141	1.000000	3.320117	6.685894
**0**	**3**	**2**	1.000000	1.045673	1.099286	1.000000	1.221403	1.284025
**0**	**4**	**1**	1.000000	0.818731	0.449329	1.000000	0.818731	0.449329
**0**	**5**	**0**	1.000000	1.000000	1.000000	1.000000	1.000000	1.000000
**1**	**0**	**4**	139.191054	1.000000	139.191054	1096.633163	1.000000	1096.633163
**1**	**1**	**3**	23.051245	8.480079	19.840390	73.699794	27.112639	63.434000
**1**	**2**	**2**	5.298386	3.213633	3.925141	9.025014	5.473947	6.685894
**1**	**3**	**1**	1.724023	1.724023	1.099286	2.013753	2.013753	1.284025
**1**	**4**	**0**	0.818731	1.349859	1.000000	0.818731	1.349859	1.000000
**2**	**0**	**3**	139.191054	1.000000	139.191054	1096.633163	1.000000	1096.633163
**2**	**1**	**2**	23.051245	13.981287	19.840390	73.699794	44.701185	63.434000
**2**	**2**	**1**	5.298386	5.298386	3.925141	9.025014	9.025014	6.685894
**2**	**3**	**0**	1.724023	2.842434	1.000000	2.013753	3.320117	1.000000
**3**	**0**	**2**	139.191054	1.000000	139.191054	1096.633163	1.000000	1096.633163
**3**	**1**	**1**	23.051245	23.051245	19.840390	73.699794	73.699794	63.434000
**3**	**2**	**0**	5.298386	8.735561	1.000000	9.025014	14.879732	1.000000
**4**	**0**	**1**	139.191054	1.000000	139.191054	1096.633163	1.000000	1096.633163
**4**	**1**	**0**	23.051245	38.005077	1.000000	73.699794	121.510418	1.000000
**5**	**0**	**0**	139.191054	1.000000	1.000000	1096.633163	1.000000	1.000000

Table S3A. The relative payoffs of punishers to cooperators with $\alpha = 1.0 \sim 2.0$.

N_x	N_y	N_z	P_z/P_x ($\alpha = 1.0$)	P_z/P_x ($\alpha = 1.2$)	P_z/P_x ($\alpha = 1.4$)	P_z/P_x ($\alpha = 1.6$)	P_z/P_x ($\alpha = 1.8$)	P_z/P_x ($\alpha = 2.0$)
**0**	**0**	**5**						
**0**	**1**	**4**						
**0**	**2**	**3**						
**0**	**3**	**2**						
**0**	**4**	**1**						
**0**	**5**	**0**						
**1**	**0**	**4**	1	1	1	1	1	1
**1**	**1**	**3**	0.785714286	0.861548195	0.905620094	0.933536447	0.952194583	0.965116279
**1**	**2**	**2**	0.25	0.517030974	0.665425401	0.758037947	0.820079404	0.863636364
**1**	**3**	**1**	-3.5	-1.378196529	-0.542655735	-0.099089139	0.173797522	0.357142857
**1**	**4**	**0**	0	0	0	0	0	0
**2**	**0**	**3**	1	1	1	1	1	1
**2**	**1**	**2**	0.785714286	0.861548195	0.905620094	0.933536447	0.952194583	0.965116279
**2**	**2**	**1**	0.25	0.517030974	0.665425401	0.758037947	0.820079404	0.863636364
**2**	**3**	**0**	0	0	0	0	0	0
**3**	**0**	**2**	1	1	1	1	1	1
**3**	**1**	**1**	0.785714286	0.861548195	0.905620094	0.933536447	0.952194583	0.965116279
**3**	**2**	**0**	0	0	0	0	0	0
**4**	**0**	**1**	1	1	1	1	1	1
**4**	**1**	**0**	0	0	0	0	0	0
**5**	**0**	**0**	0	0	0	0	0	0
**Average**			0.223809524	0.416034	0.500337023	0.54783974	0.578036005	0.598650961

Table S3B. The relative payoffs of cooperators to defectors with $\alpha = 1.0 \sim 2.0$ **.**

N_x	N_y	N_z	P_x/P_y ($\alpha = 1.0$)	P_x/P_y ($\alpha = 1.2$)	P_x/P_y ($\alpha = 1.4$)	P_x/P_y ($\alpha = 1.6$)	P_x/P_y ($\alpha = 1.8$)	P_x/P_y ($\alpha = 2.0$)
**0**	**0**	**5**						
**0**	**1**	**4**	0	0	0	0	0	0
**0**	**2**	**3**	0	0	0	0	0	0
**0**	**3**	**2**	0	0	0	0	0	0
**0**	**4**	**1**	0	0	0	0	0	0
**0**	**5**	**0**						
**1**	**0**	**4**						
**1**	**1**	**3**	-2.333333333	12.98904303	2.696867077	1.795623402	1.467788163	1.303030303
**1**	**2**	**2**	-4	5.126847893	2.260522157	1.675800022	1.428301397	1.294117647
**1**	**3**	**1**	1	1	1	1	1	1
**1**	**4**	**0**	-0.666666667	-0.666666667	-0.666666667	-0.666666667	-0.666666667	-0.666666667
**2**	**0**	**3**						
**2**	**1**	**2**	3.5	1.857030964	1.459001377	1.284596059	1.189557666	1.131578947
**2**	**2**	**1**	1	1	1	1	1	1
**2**	**3**	**0**	0.166666667	0.274541197	0.368451431	0.450205037	0.521375685	0.583333333
**3**	**0**	**2**						
**3**	**1**	**1**	1	1	1	1	1	1
**3**	**2**	**0**	0.444444444	0.554032466	0.642003325	0.71262119	0.769309085	0.814814815
**4**	**0**	**1**						
**4**	**1**	**0**	0.583333333	0.684225715	0.760687843	0.818635299	0.862551259	0.895833333
**5**	**0**	**0**						
**Average**			0.049603175	1.701361043	0.751490467	0.64791531	0.612301185	0.596860122

Table S3C. The relative payoffs of punishers to defectors with $\alpha = 1.0 \sim 2.0$.

N_x	N_y	N_z	P_z/P_y ($\alpha = 1.0$)	P_z/P_y ($\alpha = 1.2$)	P_z/P_y ($\alpha = 1.4$)	P_z/P_y ($\alpha = 1.6$)	P_z/P_y ($\alpha = 1.8$)	P_z/P_y ($\alpha = 2.0$)
**0**	**0**	**5**						
**0**	**1**	**4**	-0.6875	-2.240592325	16.11396739	2.783647397	1.824316854	1.482142857
**0**	**2**	**3**	-0.166666667	-0.847735243	-5.773823164	3.918322323	2.048842785	1.583333333
**0**	**3**	**2**	0.875	0.839114997	0.759956124	0.447941186	2.11955314	1.25
**0**	**4**	**1**	4	4	4	4	4	4
**0**	**5**	**0**						
**1**	**0**	**4**						
**1**	**1**	**3**	-1.833333333	11.19068658	2.442337016	1.676279891	1.397619939	1.257575758
**1**	**2**	**2**	-1	2.650739157	1.504208863	1.270320009	1.171320559	1.117647059
**1**	**3**	**1**	-3.5	-1.378196529	-0.542655735	-0.099089139	0.173797522	0.357142857
**1**	**4**	**0**	0	0	0	0	0	0
**2**	**0**	**3**						
**2**	**1**	**2**	2.75	1.599921675	1.321300964	1.199217241	1.132690366	1.092105263
**2**	**2**	**1**	0.25	0.517030974	0.665425401	0.758037947	0.820079404	0.863636364
**2**	**3**	**0**	0	0	0	0	0	0
**3**	**0**	**2**						
**3**	**1**	**1**	0.785714286	0.861548195	0.905620094	0.933536447	0.952194583	0.965116279
**3**	**2**	**0**	0	0	0	0	0	0
**4**	**0**	**1**						
**4**	**1**	**0**	0	0	0	0	0	0
**5**	**0**	**0**						
**Average**			0.105229592	1.228036963	1.528309782	1.20630095	1.117172511	0.997764269

Table S4A. The relative fitnesses of punishers to cooperators with $\alpha = 1.0 \sim 2.0$.

N_x	N_y	N_z	F_z/F_x ($\alpha = 1.0$)	F_z/F_x ($\alpha = 1.2$)	F_z/F_x ($\alpha = 1.4$)	F_z/F_x ($\alpha = 1.6$)	F_z/F_x ($\alpha = 1.8$)	F_z/F_x ($\alpha = 2.0$)
**0**	**0**	**5**	2.718282	4.8046989	10.543198	31.180464	139.19106	1096.6332
**0**	**1**	**4**	1.733253	2.543165	4.2178321	8.2225142	19.840389	63.433998
**0**	**2**	**3**	1.105171	1.378723	1.816045	2.559633	3.9251411	6.685894
**0**	**3**	**2**	0.70468801	0.77045	0.853598	0.96024197	1.099286	1.284025
**0**	**4**	**1**	0.44932899	0.44932899	0.44932899	0.44932899	0.44932899	0.44932899
**0**	**5**	**0**	1	1	1	1	1	1
**1**	**0**	**4**	1	1	1	1	1	1
**1**	**1**	**3**	0.86070787	0.86070774	0.86070792	0.86070795	0.86070793	0.86070798
**1**	**2**	**2**	0.74081811	0.74081801	0.74081831	0.74081826	0.74081825	0.74081814
**1**	**3**	**1**	0.63762805	0.63762824	0.6376282	0.63762822	0.63762837	0.63762785
**1**	**4**	**0**	1.2214024	1.2214024	1.2214024	1.2214024	1.2214024	1.2214024
**2**	**0**	**3**	1	1	1	1	1	1
**2**	**1**	**2**	0.86070787	0.86070774	0.86070792	0.86070795	0.86070793	0.86070798
**2**	**2**	**1**	0.74081811	0.74081801	0.74081831	0.74081826	0.74081825	0.74081814
**2**	**3**	**0**	0.90483738	0.82760496	0.74698886	0.66402869	0.58003867	0.49658525
**3**	**0**	**2**	1	1	1	1	1	1
**3**	**1**	**1**	0.86070787	0.86070774	0.86070792	0.86070795	0.86070793	0.86070798
**3**	**2**	**0**	0.67031992	0.53732185	0.40792948	0.28942362	0.18873672	0.11080315
**4**	**0**	**1**	1	1	1	1	1	1
**4**	**1**	**0**	0.49658525	0.3384396	0.20406405	0.10467698	0.04338161	0.01356856
**5**	**0**	**0**	0.36787942	0.20812959	0.09484789	0.03207136	0.00718437	0.00091188
**Average**			0.95586358	1.0847929	1.4407916	2.6373892	8.3927301	56.191956

Table S4B. The relative fitnesses of cooperators to defectors with $\alpha = 1.0 \sim 2.0$.

N_x	N_y	N_z	F_x/F_y ($\alpha = 1.0$)	F_x/F_y ($\alpha = 1.2$)	F_x/F_y ($\alpha = 1.4$)	F_x/F_y ($\alpha = 1.6$)	F_x/F_y ($\alpha = 1.8$)	F_x/F_y ($\alpha = 2.0$)
**0**	**0**	**5**	1	1	1	1	1	1
**0**	**1**	**4**	2.225541	1.516781	0.914552	0.46913	0.194423	0.06081
**0**	**2**	**3**	1.822118	1.460592	1.108868	0.786735	0.51304	0.301194
**0**	**3**	**2**	1.491825	1.364491	1.231577	1.094799	0.956322	0.818731
**0**	**4**	**1**	1.221402	1.221402	1.221402	1.221402	1.221402	1.221402
**0**	**5**	**0**	1	1	1	1	1	1
**1**	**0**	**4**	2.718282	4.804699	10.5432	31.18046	139.1911	1096.633
**1**	**1**	**3**	2.718283	2.718282	2.718281	2.718282	2.718282	2.718282
**1**	**2**	**2**	1.648722	1.648722	1.648721	1.648721	1.648722	1.648722
**1**	**3**	**1**	1	1	1	1	1	1
**1**	**4**	**0**	0.606531	0.606531	0.606531	0.606531	0.606531	0.606531
**2**	**0**	**3**	2.718282	4.804699	10.5432	31.18046	139.1911	1096.633
**2**	**1**	**2**	1.648721	1.648722	1.648721	1.648721	1.648721	1.648721
**2**	**2**	**1**	1	1	1	1	1	1
**2**	**3**	**0**	0.606531	0.606531	0.606531	0.606531	0.606531	0.606531
**3**	**0**	**2**	2.718282	4.804699	10.5432	31.18046	139.1911	1096.633
**3**	**1**	**1**	1	1	1	1	1	1
**3**	**2**	**0**	0.606531	0.606531	0.606531	0.606531	0.606531	0.606531
**4**	**0**	**1**	2.718282	4.804699	10.5432	31.18046	139.1911	1096.633
**4**	**1**	**0**	0.606531	0.606531	0.606531	0.606531	0.606531	0.606531
**5**	**0**	**0**	2.718282	4.804699	10.5432	31.18046	139.1911	1096.633
**Average**			1.609245	2.048981	3.363535	8.234106	33.91821	261.8576

Table S4C. The relative fitnesses of punishers to defectors with $\alpha = 1.0 \sim 2.0$.

N_x	N_y	N_z	F_z/F_y ($\alpha = 1.0$)	F_z/F_y ($\alpha = 1.2$)	F_z/F_y ($\alpha = 1.4$)	F_z/F_y ($\alpha = 1.6$)	F_z/F_y ($\alpha = 1.8$)	F_z/F_y ($\alpha = 2.0$)
**0**	**0**	**5**	2.718282	4.804699	10.5432	31.18046	139.1911	1096.633
**0**	**1**	**4**	3.857425	3.857424	3.857425	3.857425	3.857425	3.857425
**0**	**2**	**3**	2.013752	2.013752	2.013753	2.013753	2.013753	2.013753
**0**	**3**	**2**	1.051271	1.051272	1.051272	1.051272	1.051271	1.051271
**0**	**4**	**1**	0.548811	0.548811	0.548811	0.548811	0.548811	0.548811
**0**	**5**	**0**	1	1	1	1	1	1
**1**	**0**	**4**	2.718282	4.804699	10.5432	31.18046	139.1911	1096.633
**1**	**1**	**3**	2.339648	2.339646	2.339646	2.339647	2.339647	2.339647
**1**	**2**	**2**	1.221403	1.221403	1.221403	1.221403	1.221403	1.221403
**1**	**3**	**1**	0.637628	0.637628	0.637628	0.637628	0.637628	0.637628
**1**	**4**	**0**	0.740818	0.740818	0.740818	0.740818	0.740818	0.740818
**2**	**0**	**3**	2.718282	4.804699	10.5432	31.18046	139.1911	1096.633
**2**	**1**	**2**	1.419067	1.419068	1.419068	1.419068	1.419067	1.419068
**2**	**2**	**1**	0.740818	0.740818	0.740818	0.740818	0.740818	0.740818
**2**	**3**	**0**	0.548812	0.501968	0.453072	0.402754	0.351811	0.301194
**3**	**0**	**2**	2.718282	4.804699	10.5432	31.18046	139.1911	1096.633
**3**	**1**	**1**	0.860708	0.860708	0.860708	0.860708	0.860708	0.860708
**3**	**2**	**0**	0.40657	0.325902	0.247422	0.175544	0.114475	0.067206
**4**	**0**	**1**	2.718282	4.804699	10.5432	31.18046	139.1911	1096.633
**4**	**1**	**0**	0.301194	0.205274	0.123771	0.06349	0.026312	0.00823
**5**	**0**	**0**	1	1	1	1	1	1
**Average**			1.537111	2.023238	3.3796	8.284546	33.99425	261.9511

Table S5. The average relative fitnesses of punishers to cooperators with different punishers amount in N with $\alpha = 1.0 \sim 2.0$.

N_z	**Average** F_z/F_y ($\alpha = 1.0$)	**Average** F_z/F_y ($\alpha = 1.2$)	**Average** F_z/F_y ($\alpha = 1.4$)	**Average** F_z/F_y ($\alpha = 1.6$)	**Average** F_z/F_y ($\alpha = 1.8$)	**Average** F_z/F_y ($\alpha = 2.0$)
**0**	0.77683739	0.6888164	0.61253878	0.55193384	0.50679063	0.47387854
**1**	0.73769661	0.73769661	0.73769669	0.73769668	0.73769671	0.73769659
**2**	0.82655349	0.84299394	0.86378106	0.89044204	0.92520304	0.97138777
**3**	0.98862628	1.0798103	1.2255843	1.473447	1.9286163	2.8488673
**4**	1.3666265	1.7715825	2.608916	4.6112571	10.420195	32.216999
**5**	2.718282	4.8046989	10.543198	31.180464	139.19106	1096.6332

The calculation of the data in this table is based in Table S2a. The average F_z/F_y is the average relative fitness value among all the combinations where N_z is specified.
